# Pathways to bioeconomy development: A multi-regional perspective from Europe

**DOI:** 10.1007/s13280-025-02297-4

**Published:** 2025-12-03

**Authors:** Siebe Briers, Anne Ackermann, Ivana Živojinović, Stefanie Linser, Radek Rinn, Inazio Martinez de Arano, Johanna Klapper, Venla Wallius, Melanie Amato Kriján, Leire Barañano Orbe, Míriam Gonzalez Dominguez, Sari Koivula, Gudrun Van Langenhove, Stefanie Wieland

**Affiliations:** 1 European Forest Institute, Yliopistokatu 6B, 80100 Joensuu, Finland; 2 Institute of Forest, Environmental and Natural Resource Policy, BOKU University, Feistmantelstrasse 4, 1180 Vienna, Austria; 3 European Forest Institute, Forest Policy Research Network, Feistmantelstrasse 4, 1180 Vienna, Austria; 4 Centre for Bioeconomy, BOKU University, Peter-Jordan-Straße 82/II, 1190 Vienna, Austria; 5 Faculty of Forestry and Wood Sciences, Czech University of Life Sciences Prague, Kamýcká 129, 165 00 Praha–Suchdol, Czech Republic; 6 Cesefor Foundation, Pol. Ind. Las Casas, Calle C, 42005 Soria, Spain; 7 NEIKER - Basque Institute of Agricultural Research and Development, Parque Tecnológico de Bizkaia, P.812, Berreaga 1, 48160 Derio, Spain; 8 Ministry of Climate Action, Food and Rural Agenda, Government of Catalonia, Gran Via de Les Corts Catalanes, 612-614, 08007 Barcelona, Catalonia Spain; 9 Regional Council of North Karelia, Siltakatu 2, 80100 Joensuu, Finland; 10 Agency for Nature and Forests of the Flemish Government, Koning Albert II-Laan 15 Bus 177, 1210 Brussels, Belgium; 11 Wald und Holz NRW – Centre of Forest and Wood Industry (FB V), Team Wood-Based Industries, Carlsauestr. 91a, 59939 Olsberg, Germany

**Keywords:** Bioeconomy perceptions, Bioeconomy transition, Circular economy, Private sector, Public sector, Regional bioeconomy

## Abstract

**Abstract:**

The transition towards a sustainable future is increasingly understood to rely on further development of the bioeconomy. In this, both public and private sectors play pivotal roles. Government agencies and public institutions are instrumental in shaping the trajectory of the bioeconomy through strategic frameworks, regulatory measures, and policies. These instruments may create a conducive environment by clearing away bureaucratic impediments and establishing favourable conditions. Concurrently, private sector entities, including industry interest groups and companies, have the important task of advocating for these favourable conditions and driving the bioeconomy’s growth through active involvement, strategic business decisions, capital investments, and bringing bio-based innovations to market. Throughout these processes, perceptions of the bioeconomy held by actors in both sectors shape the outcomes of their actions. Hence, this study delves into the perceptions of the bioeconomy among stakeholders from both the public and private sectors across nine European regions regarding barriers and supporting conditions impacting its development, particularly important bioeconomy value chains, and the willingness and perceived responsibility to advance the bioeconomy. Findings from 534 online survey responses (288 public sector and 246 private sector) reveal that key factors identified as propelling the development of the bioeconomy forward include access to investment and scientific knowledge, while obstacles such as limited cooperation among stakeholders and inadequate supportive policies and legislative environments were noted as primary hindrances. Among the value chains highlighted, bioenergy was frequently recognised as having high growth potential, while not necessarily being the one with the most significant environmental benefits. Both the public and the private sector demonstrated a high willingness to develop the bioeconomy, yet both also assigned more responsibility to the public sector in three main areas: enhancing societal awareness and communication about the bioeconomy, ensuring beneficial environmental and social impacts, and investing in the bioeconomy's growth.

**Graphical abstract:**

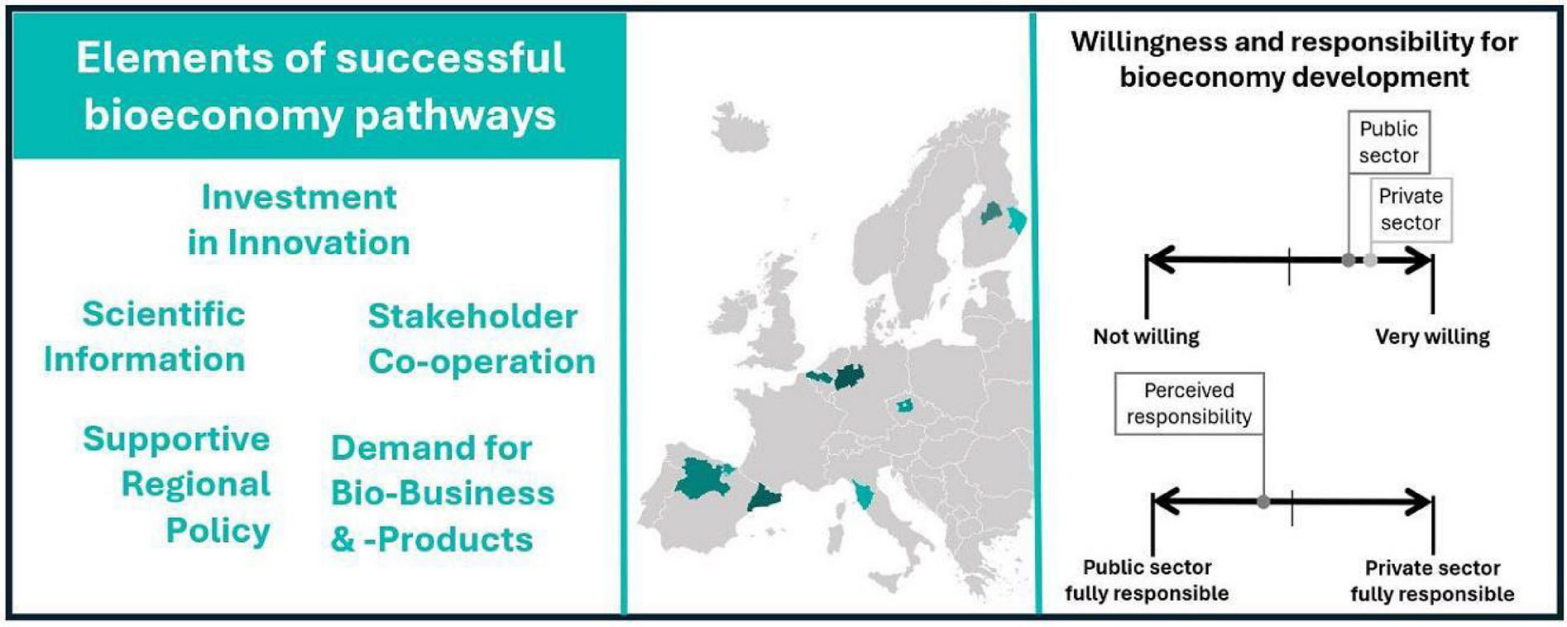

**Supplementary Information:**

The online version contains supplementary material available at 10.1007/s13280-025-02297-4.

## Introduction

Given the high hopes and aspirations attached to the possibility of sustainable economic systems, the so-called bioeconomy has become the focus of many political and policy endeavours. In the European context, the European Commission's Bioeconomy Strategy outlines how to promote the bioeconomy (European Commission 2018). Furthermore, numerous policy and strategy documents at global, European, national, and regional levels outline ambitions for the bioeconomy: 194 regions in the EU-27 have or are working towards a strategic framework related to the bioeconomy, according to Haarich and Kirchmayr-Novak (2022). Of these, 29 regions had or were working towards fully dedicated bioeconomy strategies. Sixty-nine regions had or were working towards strategic frameworks with strong bioeconomy focus. Lastly, 96 regions had or were working towards strategies with minimal bioeconomy content. To set the scene, we first summarise the extent to which the nine study regions that are the focus of this paper had progressed in formalising the bioeconomy agenda by the end of 2022 (Table S1). These regions were selected from the networks of the European Forest Institute’s (EFI) Bioregions Facility and the European Regions for Innovation in Agriculture, Food and Forestry (ERIAFF). The governments of these nine regions are actively working to advance the bioeconomy within their territories, making these regions particularly relevant objects of study for insight generation.

We notice the uneven but steadily widening policy uptake of the bioeconomy concept across Europe and underlines why stakeholders may interpret “bioeconomy” through very different strategic lenses. This overview is given in supplementary material Table S1, which indicates that only four regions have stand-alone bioeconomy strategies; in some regions the agenda was advanced via circular economy or sector-specific plans. This heterogeneity is a useful backdrop when interpreting the data presented in the following sections.

Yet, the critical challenge remains: how will these frameworks be filled with substantive meaning and translated into concrete action? To inform this, the following question needs to be answered: How can the bioeconomy develop and operate most effectively? This paper aims to contribute to answering this question.

Multiple definitions of the bioeconomy coexist, including those emphasising sustainability, resource efficiency, and innovation-driven development (McCormick and Kautto 2013; Bugge et al. 2016; Bryden et al. 2017; Global Bioeconomy Summit 2018; Frisvold et al. 2021; Töller et al. 2021; Sinkko et al. 2023). Systematic mapping studies identify at least three dominant ‘visions’ of the bioeconomy concept: the biotechnology, bioresource, and bioecology visions, each mobilising contrasting narratives of economic growth, fossil substitution, and ecological regeneration (Bugge et al. 2016; Birner 2018, 2021). One interpretation that is widely used and adopted here describes the bioeconomy as the ‘knowledge-based production and utilisation of biological resources, biological processes, and principles to sustainably provide goods and services across all economic sectors’ (IACGB 2023, p.1). However, studies have shown that the bioeconomy’s conceptual imprecision, particularly the lack of clearly defined objectives, vague definitions, and terminology, can hinder effective policy-making and stakeholder engagement (Greer 2022; Gardossi et al. 2023). Research also emphasises the value of exploring stakeholders’ perceptions of the bioeconomy’s societal and environmental dimensions. Despite the dominance of technoscientific and economic narratives in the bioeconomy discourse, these narratives face criticism for failing to deliver anticipated social and ecological benefits (Giuntoli et al. 2023). By explicitly foregrounding the contested nature of the term, our study seeks to reveal how different stakeholder groups understand bioeconomy and what this means for the transformative ambitions attached to the bioeconomy agenda.

Aside from more or less successful attempts at conceptual precision, a broad consensus on its meaning, components, and priorities appears to be emerging among practitioners, emphasising a shift towards bio-based systems that balance ecological, social, and economic objectives (Briers et al. 2024). However, the success of an effective establishment of the bioeconomy across Europe critically relies on the active involvement and strategic choices of regional stakeholders who are optimally positioned to tailor solutions that address their unique local needs, challenges, and opportunities (Gerdes and Kiresiewa 2018; D’Amato et al. 2020; Gardossi et al. 2023; Briers et al. 2024). Hence, a definitional focus should be complemented by one that identifies key questions that determine the success of bioeconomic strategies and practices.

One crucial question is: where should attention and efforts be focused? High-growth-potential value chains, which offer promising pathways for both sustainability and economic development, warrant particular focus. Another essential question is: what works and what does not, and what are the bioeconomy’s most important barriers and supporting conditions in diverse contexts. Finally, to enable the most effective implementation of the bioeconomy, we must determine who the most suitable actors are, which actors different stakeholders see as responsible for this societal project, and which actors are best placed to shape it constructively. First, answers to all these questions—admittedly imperfect and incomplete, but nevertheless a helpful practical indication and a strong basis for further academic engagement—will be offered in the present paper based on a survey of the perceptions of representatives of the public and private sectors in nine European regions.

These sectors are among the most impactful actors in shaping the bioeconomy. It is true that diverse other stakeholders are also involved in the shaping of the bioeconomy: local communities, research institutions, environmental organisations, among others, and according to the principles of ‘systems thinking’, incorporating the viewpoints of these diverse stakeholders is essential for cultivating favourable perceptions and outcomes of bioeconomic initiatives (Meadows 2008). However, the public and private sectors have a central role and constitute the focus of this paper.

The private sector primarily influences the bioeconomy’s direction through strategic business decisions, investments, and innovations and by lobbying for advantageous conditions. This intersects with the roles of public institutions and governance structures, which are equipped to shape these conditions through strategies, policy-making, and regulatory measures. Such governance efforts ensure broad and lasting benefits and foster an environment conducive to bioeconomy stakeholders. Robust governance measures are crucial for safeguarding economic, social, and ecological sustainability and for enabling fair competitive conditions for bioeconomy products and processes, thus making efficient market choices between alternative and traditional technologies and resources viable (Thran and Moesenfechtel 2022).

However, a deeper comprehension of the perceptions held by public and private sector actors on prevailing barriers and supporting conditions, as well as ways to get involved in the bioeconomy, can aid practitioners and policy makers in enhancing the bioeconomy's cohesion and efficiency through collaborative and synergistic efforts. This helps in understanding the dynamics that either hinder or facilitate the bioeconomy and in identifying regionally specific priorities for bioeconomy initiatives. Insights from such an analysis are offered here based on a survey in nine European regions (cf. Government and Industry’s Bioeconomy Perceptions Survey (Bioregions Facility 2024) for regional context) and may inform future strategies and policies in these regions—and potentially broader governance levels.

Building on previous research (Briers et al. 2024), this paper seeks to address the following questions: (1) How do respondents’ demographic traits (sector affiliation, regional background) correlate with their self-assessed familiarity with the bioeconomy? (2) How do respondents assess public information levels about the bioeconomy? (3) Which bioeconomy value chains are perceived to have a high growth potential? (4) What factors and dynamics are considered the main barriers and supporting conditions to bioeconomy development? (5) What roles and responsibilities do the public and private sectors have in the transition towards a bioeconomy? (6) How willing are both surveyed groups to develop the bioeconomy? Providing policy makers and relevant practitioners with empirically based insights from diverse regions across Europe, this paper aims to improve their understanding of relevant actors’ perceptions of where there might be additional information needs, what currently works well or not in the bioeconomy, what are its immediate potentials, what are and should be their own and others’ role in it—in short: it aims to equip policy makers and other relevant actors to optimally develop the bioeconomy for the benefit of societies in different geographical contexts, at different levels of political support and advancement of the bioeconomy, and working with different bioeconomic opportunities and constraints.

The paper is organised as follows: Sect. "Literature on dynamics of bioeconomy development" provides insights from scholarly literature on the dynamics of bioeconomy development and on bioeconomy perceptions. Sect. "Methods" details the methods employed in the study. Sect. "Results" presents the results, structured into three subsections: Bioeconomy familiarity and information levels; Key characteristics of the bioeconomy and its development; Willingness and responsibility to develop the bioeconomy. Sect. "Discussion" discusses the findings, while Sect. "Conclusion" concludes the paper, offering recommendations and directions for future studies.

## Literature on dynamics of bioeconomy development

Much academic and non-academic discussion focuses on the opportunities associated with the bioeconomy. For instance, bioeconomy strategies align well with the Sustainable Development Goals, fostering synergies among clean energy, recycling, and ecosystem preservation (Ronzon and Sanjuán, 2020). It includes public–private partnerships and EU-funded projects that drive innovation and the development of new bio-based products and materials (Open Access Government 2023) and has significant potential for economic growth, job creation, and rural development, enhancing resilience to climate change and promoting sustainability (Lange et al. 2021).

The question then arises: what hinders or advances successful bioeconomy development and, ultimately, the achievement of these opportunities? The literature suggests that potential barriers and supporting conditions for bioeconomy development are very diverse. Key themes include governance, investment, and awareness issues.

### Governance as a factor in bioeconomy development

Governance aspects emerge as a recurring theme in bioeconomy literature emphasising its dual role as a driver and a challenge in the bioeconomy.

Proestou et al. (2024) conducted an analysis of policy documents related to the bioeconomy, examining the relationship between economic and environmental objectives. Government bioeconomy strategies, analysed through 78 policy documents from 50 countries, reveal a predominance of economic objectives focused on market development, biomass management and sustainable growth. Environmental objectives, while present, are often aligned with economic perspectives and reflect a trend towards framing bioeconomy strategies in terms of sustainability and green growth, particularly in high-income countries. Policy objectives focus on governance and regulation, while social objectives, such as addressing inequalities, remain less prominent. Additionally, Dietz et al. (2018) conducted a global comparative analysis of 41 national bioeconomy strategies, identifying enabling and constraining governance mechanisms critical to aligning bioeconomy initiatives with Sustainable Development Goals (SDGs). Their findings highlight the importance of strategic coordination to ensure sustainability is embedded in governance frameworks. Heimann (2018) showed in his study that without additional measures and efforts (regulations, policies, and investments ensuring sustainability), the bioeconomy has the potential to limit rather than support the achievement of the SDGs. Ferraz and Pyka (2023), through their systematic literature review, highlight the bioeconomy’s potential to contribute to achieving the SDGs. However, additional research is essential to design and implement bioeconomy policies that bridge these gaps and advance sustainable development.

Stern et al. (2018) highlight the necessity of inclusive governance frameworks that address societal perceptions and public trust in bioeconomic initiatives. This highlights the need for comprehensive frameworks that balance competing interests while fostering societal trust in bioeconomy advancements. Dietz et al. (2023) highlight significant governance gaps at both national and international levels in advancing the bioeconomy and offer expert-recommended solutions. At the national level, experts emphasise insufficient policy coordination. At the international level, they highlight the lack of binding regulations, unequal distribution of institutional capacities, and imbalances in knowledge and technology access. The current governance frameworks contain incoherent policy incentives. De Besi and McCormick (2015) call for coherent bioeconomy strategies. Banda and Huzair (2021) emphasise that governance frameworks need to address tensions arising from regulatory practices, particularly balancing precautionary approaches with industry interests, including the drive for innovation. Enhanced global and cross-sectoral policy coordination is essential to steer the bioeconomy towards sustainable development and minimise risks to social and environmental sustainability.

Focusing on specific policy incoherences, the risk of conflicts between SDGs in bioeconomy strategies is highlighted, particularly concerning forest-based trade-offs (Maksymiv et al. 2021). Their analysis calls for governance mechanisms that mitigate these tensions to achieve coherence across policy goals. Mustalahti (2018) introduces the concept of responsive governance, emphasising citizen inclusion as crucial for equitable and adaptive governance in bioeconomy transitions. Strategic coordination and responsive governance frameworks ensure alignment with the Sustainable Development Goals, addressing competing interests and embedding sustainability principles into bioeconomic growth (Dietz et al. 2018; Stern et al. 2018).

Gawel et al. (2018) underline the importance of creating fair competitive conditions through harmonised policies to facilitate the transition towards the bioeconomy. This aligns with broader discussions around creating a level playing field for new bio-based activities while maintaining environmental integrity. Dieken et al. (2021) extend this concern, highlighting that stakeholders' perceptions of the bioeconomy are predominantly technology- and resource-oriented, with limited attention to its ecological dimension. The study underscores the lack of public involvement, challenging the bioeconomy’s claim to contribute to sustainable development. To address these gaps, it calls for comprehensive strategies that integrate diverse stakeholder perspectives beyond narrow consumption topics, fostering inclusive governance structures and ensuring a balanced approach to sustainability. Similarly, Paşnicu et al. (2019) stress the importance of cohesive governance and harmonised policy approaches to drive sustainable bioeconomy development. Concrete examples include the demand for government intervention to support planning security, essential for long-term investments in sectors like the wood-based bioeconomy (Hafner et al. 2020). Similarly, Franzini et al. (2018) underscore the influence of local governments in promoting sustainable construction practices, such as the use of wood in multi-storey buildings. These findings emphasise the role of local governments as critical gatekeepers in urban planning and their ability to influence sustainable construction practices.

This body of research underscores the multifaceted governance challenges in the bioeconomy and highlights the importance of inclusive, strategic, and well-coordinated policies.

### Investment and public spending

Finance, particularly investment in research, development, and innovation, is also a key consideration in factors that promote or hinder bioeconomy development. Economic hurdles, such as limited access to capital for bioeconomy start-ups and inadequate commercialisation support, hinder progress (Dietz et al. 2018; Faulkner et al. 2024; Hogarth and Salter 2010). Other authors have found that public spending on research and development has proved helpful in the development of advanced bioeconomy sectors, for example, biorefineries (Ding and Grundmann 2021). Albrecht et al. (2021) highlight the potential economic opportunities for industrial and technological development as a dominant narrative that justifies public finance for the bioeconomy. EU-supported initiatives, such as the Circular Bio-based Europe Joint Undertaking, significantly enhance competitiveness and resilience by funding innovative bio-based products, facilities, and processes, while promoting rural development and sustainability (Open Access Government 2023; CBE JU 2024). Prochaska and Schiller (2024) demonstrated, using Germany as an example, that expanding research and development funding for biological activities from a biotechnological focus to encompass bioresources and bioecology significantly enhances the participation of rural and less-developed regions. This shift is driven by the inclusion of more traditional industrial sectors as key recipients of research and development support, highlighting the potential for regional development through diversified bioeconomy initiatives.

### Public perceptions and engagement in the bioeconomy

Public perceptions and engagement are critical for the success of bioeconomy strategies, as societal acceptance and active participation influence the long-term sustainability of bioeconomic transitions—not least in terms of attracting a qualified workforce (Pender et al. 2024). Hence, several authors observe a need to improve public awareness of the bioeconomy and its benefits (Dieken and Venghaus 2020; Dupont-Inglis and Borg 2018; Pascoli et al. 2021; Thomchick et al. 2024). Macht et al. (2022) show that the perception of a bioeconomy transition depends on the specific technologies that will be implemented and on how these technologies are communicated to the public.

Pasnicu et al. (2019) and Ranacher et al. (2020) point to the general population's limited understanding of bioeconomy concepts, stressing the need for targeted awareness campaigns to bridge knowledge gaps. For instance, the Austrian case study by Stern et al. (2018) reveals that public trust in bioeconomy projects is closely tied to transparent communication about their environmental and economic impacts. These findings are echoed in broader European contexts, where public awareness campaigns have been shown to foster community-level engagement with bio-based practices. Woźniak et al. (2020) further emphasise that effective communication of bioeconomy benefits—especially regarding sustainability and economic growth—is key to public acceptance across Europe. Equally, education significantly influences perceptions of the bioeconomy by raising awareness, shaping sectoral importance, and providing interdisciplinary and transformative knowledge (Paris et al. 2023; Marcineková et al. 2023; Trigkas and Karagouni 2023; Pink et al. 2024).

Across different knowledge levels and irrespective of how knowledge is assessed, perceptions of the bioeconomy are often complex. In Austria, public perceptions reveal a divide between technology-driven visions and environmentally localised approaches, with trust in sustainable consumption emerging as a crucial factor (Stern et al. 2018). Similarly, Pasnicu et al. (2019) demonstrate that in Central and Eastern Europe, public perceptions are shaped by socioeconomic factors and national strategies, reflecting variability in readiness to engage with bioeconomy initiatives. A survey of the German population revealed limited familiarity with the term itself, but widespread support for its underlying principles, particularly those tied to sustainability and environmental benefits, though concerns persist regarding certain specific practices or technologies in value chains seen to be part of the bioeconomy (Dallendörfer et al. 2022).

Linking perceptions to behaviour, a study by Rinn et al. (2024) in countries of Southeast Asia highlights the relationship between perceptions of the bioeconomy and participation in sustainable practices, including the sustainable use of natural resources. Stern et al. (2018) and Woźniak et al. (2020) highlight that public participation in governance enhances the acceptance of bioeconomy initiatives, fostering sustainable consumer practices. Research by McCormick and Kautto (2013) underlines that participatory governance models, which actively involve citizens and stakeholders, are critical for the development of sustainable bioeconomy policies. Public engagement efforts can also draw on best practices from national bioeconomy strategies, as illustrated in studies in Finland and Germany, where stakeholder inclusion is prioritised to build trust and mitigate potential conflicts (Franzini et al. 2018; Hafner et al. 2020).

### High potential value chains in the European bioeconomy

The above-listed conditions collectively create a favourable environment for bioeconomic growth, enabling the transition towards sustainability and circularity, and the development of new bio-based products. While such structural factors are important, it is also important not to overlook the bioeconomy’s substantive content, which is also decisive for its success.

Positioned as a cornerstone for achieving sustainability, fostering innovation, and addressing climate challenges, the bioeconomy is now a significant economic sector in the EU, representing 5% of the EU's GDP and employing 8.3% of its workforce (UNECE 2024). It encompasses several high-growth-potential value chains, prominently discussed in the literature and supported by strategic EU initiatives, such as the CBE JU. Among the most prominently discussed are wood construction and bioenergy. The former has seen growing demand for engineered wood products (Franzini et al. 2018), while the latter accounts for over half of the EU's renewable energy consumption (D’Amico et al. 2021; Mastrucci et al. 2024). Long-standing value chains like wood-based materials and products, pulp and paper, non-wood forest products, and food and gastronomy are also relevant. Green chemistry and bioplastics also feature heavily. In the case of bioplastics, expanding production capacity and regulatory support foster innovative bio-based materials that align with the EU’s circular economy goals (European Commission n.d.-a). Nature-based tourism integrates economic growth with conservation, particularly in rural and ecologically sensitive areas. In addition to these widely recognised sectors, others such as sustainable textiles, focusing on bio-based and biodegradable fibres (Deckers et al. 2023), and bio-based fertilisers, which improve agricultural sustainability through organic waste conversion (Chew et al. 2019), are gaining traction. Innovations in bio-based packaging, driven by the EU’s single-use plastics directive (European Commission 2019), and in cosmetics, with natural ingredients reducing petrochemical reliance, further diversify the bioeconomy. These sectors collectively highlight the EU’s activities in fostering a circular and sustainable economy. They are supported by market insights and forecasts from sources like the EU Bioeconomy Monitoring System (European Commission, n.d.-b).

## Materials and methods

### Data collection

The research findings presented in this paper derive from an online survey conducted across nine European regions. Constituting a two-pronged approach of purposive expert sampling across the continent and convenience sampling via comparatively easily mobilised multipliers (including a snowballing effect), the selected regions were recruited as locations for sourcing survey respondents through the networks of the European Forest Institute’s (EFI) Bioregions Facility and the European Regions for Innovation in Agriculture, Food, and Forestry (ERIAFF). The choice of these specific regions followed explicit case-selection criteria intended to enable a structured, comparative exploration rather than statistical representativeness. First, regions had to (i) be active members of EFI Bioregions and/or ERIAFF and (ii) have an identified public authority willing and able to act as a local dissemination partner. Second, to maximise heterogeneity, we sought coverage across Europe’s main macro-geographies (North–South and East–West) and to certain extent varied bioeconomy profiles (forest-dominant, mixed agro-forestry, and diversified industrial bases). Third, regions were required to have ongoing policy interest in advancing bioeconomy activities (e.g. a strategy, programme, or equivalent initiative) to ensure the policy salience of stakeholder perceptions. Finally, feasibility criteria (e.g. local outreach capacity) were applied. These criteria, together with pre-existing collaboration channels in Bioregions and/or ERIAFF, explain why the final set comprises these nine regions specifically.

To glean insights from the public sector, the survey sampled stakeholders from relevant ministries, municipal departments, public agencies, and publicly owned companies. Key priority domains included were Agriculture, Energy, Environment, Forestry, Rural and Urban Development, Tourism, and Recreation. From the private sector, responses were solicited from diverse entities linked to the bioeconomy such as wood or food product manufacturers, tourism service providers, forest owners, forest managers, and representatives from industry clusters and umbrella organisations like sector/industry associations, chambers of commerce, agricultural and forestry associations, and cooperatives. The focus areas identified to instruct regional disseminators were Agri-food, Clean Tech, Environment, Forestry, Gastronomy, and Tourism. While attempting to even out imbalances in focus on different value chains by means of this instruction, it cannot be guaranteed that no such imbalances remained. We therefore treat the sample as analytical rather than probability-based: it is designed to capture breadth across relevant actor groups and value chains, not to estimate population parameters. Furthermore, an unavoidable potential selection bias in favour of commercially oriented attitudes towards the bioeconomy inherent in this sampling design was considered acceptable due to the economic focus of this research endeavour and the assessment that there were no grounds for ethical concerns about maleficent impacts of this research. This potential bias is explicitly acknowledged in the interpretation of results.

Ultimately, nine regions participated: Basque Country, Castile and León, Catalonia (Spain), North Karelia, South Ostrobothnia, (Finland), Central Bohemia (Czech Republic), Flanders (Belgium), North Rhine-Westphalia (Germany), and Tuscany (Italy). This set satisfies the heterogeneity criteria above by spanning Northern and Southern as well as Western and Central/Eastern Europe, and by including regions with distinct resource endowments and industrial structures. While a larger and more diversified set (including non-Bioregions and/or ERIAFF regions and countries beyond Europe) would further strengthen external validity, these nine cases constitute a theoretically justified and feasible comparative baseline for the present study.

Ethical considerations regarding the research subjects were reflected in the adoption of the principles of free, prior and informed consent and the option to abort participation in the research at the respondent’s discretion, as well as anonymity and GDPR conformity of the study. All steps of the research process were guided by the principles of inclusivity, fair representation, and equal value creation.

The survey tools, including a questionnaire and a dissemination toolkit, were translated into the local languages, and distributed by local partnering organisations (mentioned in the acknowledgements). The survey was available for approximately three months in each region between September 2021 and December 2022. Dissemination methods included social media and direct email campaigns. Due to the nature of these dissemination methods, accurately determining the response rate is not possible. In all regions, the local partnering organisations included a regional government agency knowledgeable about the regional bioeconomy actors. These partnering organisations disseminated the survey among the relevant public and private sector actors. The sample size was defined by the possible outreach during the three-month timeframe.

The survey included a working definition of the bioeconomy that corresponds to those referred to in the introduction, adding reference to the forest sector specifically: ‘an economy that relies on the production and utilisation of renewable biological resources, including those of forest origin, to produce materials, energy, products, and services across all economic sectors’.

During data cleaning, some of the 713 original responses were excluded from the final analysis: those providing only demographic information without substantive answers and those from participants indicating the sector (public, private, other) category ‘other’, such as researchers and media representatives. However, of these ‘others’, respondents whose additional information indicated a misunderstanding of the categories were reassigned to their corresponding sector category and included.

**Fig. 1:**
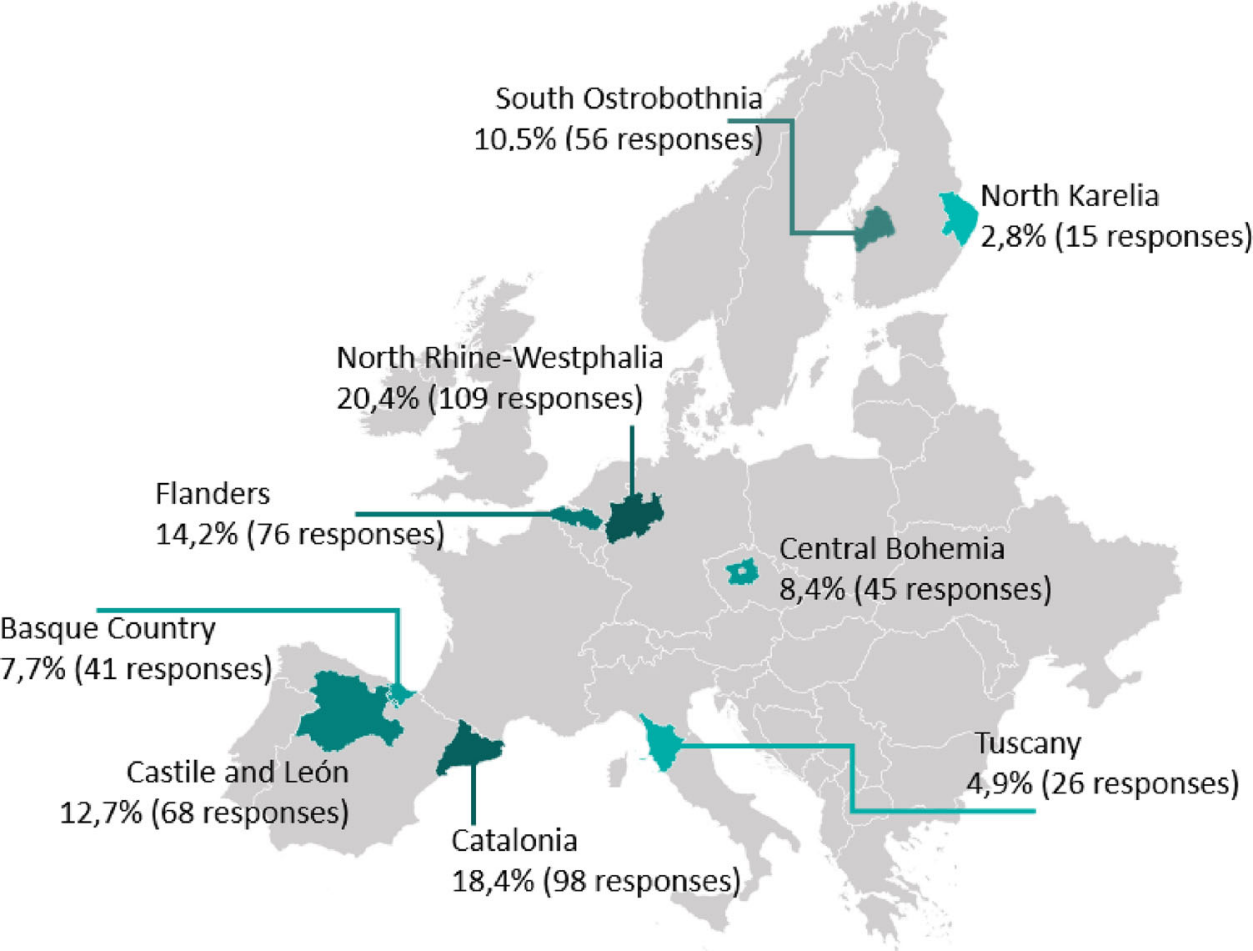
Map of regions where the survey was launched with the absolute number of respondents and as a proportion of the entire sample (Briers et al. 2024)

After processing, the final dataset included 534 responses, with 288 (53.9%) from the public sector and 246 (46.1%) from the private sector. The numbers of responses received varied significantly across regions, from 15 (2.8%) from North Karelia to 109 (20.4%) from North Rhine-Westphalia (Fig. 1). The unequal sample sizes across regions and the small sample size in a few regions represent limitations of this study. This imbalance was partly unavoidable, given that the population in North Rhine-Westphalia is more than 100 times larger than that of North Karelia. Nevertheless, the authors acknowledge these limitations and refrain from making claims of absolute representativeness. To ensure contextual accuracy, all local partner organisations reviewed the study as part of a regional validation process.

Based on open-ended responses categorising respondents' value chains, we find that the public sector is predominantly represented by forestry, followed by education, research, economic development, environmental management, and agriculture. A smaller proportion of public sector respondents are affiliated with waste management or energy-related activities. In the private sector, agriculture and forestry/wood-based industries are the most frequent, followed by the food sector, energy, and consultancy. Fewer private sector respondents operate in waste management, manufacturing, or construction.^1^

A mandatory familiarity slider from 0 to 100 was used to facilitate cross-analysis based on familiarity levels. Responses to this were later categorised as low (0–33), medium (34–66), and high (67–100) familiarity. Other questions were optional, which might have affected the total number of responses for those items.

The questionnaire inquired about following key aspects (see supplementary material 2): (1) familiarity with the bioeconomy; (2) relevant concepts, value chains,^2^ policy areas associated with the bioeconomy, and its potential benefits and risks; (3) barriers and supporting conditions for bioeconomy development; (4) high-potential value chains in the bioeconomy; (5) willingness and responsibility to advance the bioeconomy; and (6) perception of the general public’s understanding of the bioeconomy. This study reports findings about aspects 1, 3, 4, 5, and 6, while conceptual associations are discussed in Briers et al. (2024).

### Data analysis

Statistical analyses presented here were carried out using IBM SPSS Statistics, version 29. The initial step in the analysis involved employing standard descriptive and summary statistics (mean, range, and distribution) to provide a basic understanding of the data. Because the variables were not normally distributed, non-parametric methods were subsequently applied in accordance with the distribution characteristics of the data.

**Fig. 2:**
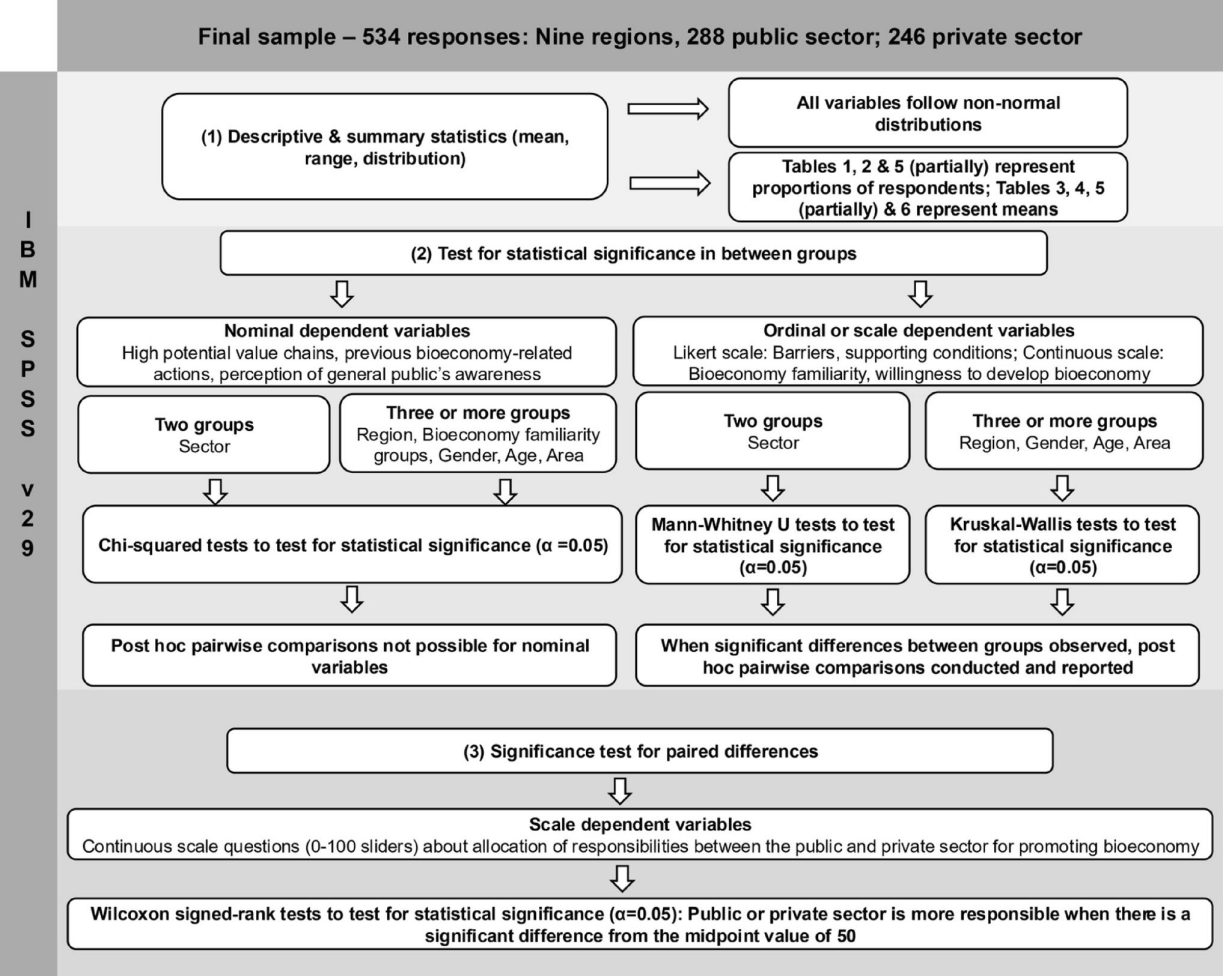
Visualisation of the data analysis methodology used

For nominal dependent variables, chi-squared tests were utilised to determine statistical significance between groups, with a significance level set at α = 0.05 (McHugh 2013; see Fig. 2). These variables included responses to multiple-choice questions regarding high-potential bioeconomy value chains and single-choice questions about respondents’ previous bioeconomy-related actions and their perceptions of the general public’s awareness.

For ordinal or scale-dependent variables, the choice of statistical test varied based on the number of groups being compared (Fig. 2). The Independent-samples Mann–Whitney U test was used to analyse differences between two groups, such as the sector variable (private vs. public sector), with the significance level set at α = 0.05 (McKnight and Najab 2010). For comparisons involving more than two groups, such as for region, bioeconomy familiarity, gender, age, and area, Kruskal–Wallis tests were applied (α = 0.05) (Cleophas and Zwinderman 2016). The ordinal or scale-dependent variables included responses to Likert scale questions assessing barriers and supporting conditions for the bioeconomy, as well as the 0–100 continuous scale (slider) questions gauging respondents' self-rated familiarity with the bioeconomy and willingness to engage in bioeconomy development.

When significant differences between groups were detected for ordinal or scale-dependent variables, post hoc pairwise comparisons were conducted and reported. This systematic approach allows for the detailed examination of differences between specific pairs of groups within the dataset (Fig. 2).

Additionally, the Wilcoxon signed-rank test was employed to analyse the 0–100 continuous scale (slider) questions concerning the allocation of responsibility for promoting the bioeconomy between the public and private sectors (Fig. 2). This test is suitable for paired differences when data distribution is non-normal (Woolson 2008). A midpoint value of 50, indicating equal responsibility attributed to both sectors, served as the reference for this analysis.

Tables 1–6 present aggregate (‘All respondents’) and compartmentalised data, covering all key groups studied (bioeconomy familiarity groups, sector, regions). Bold cells in the tables represent values with a notable difference (subjective classification by the authors) from the ‘All respondents’ value. The definition of a ‘notable’ difference is described in the table captions and does not reflect information about statistical significance.

The study also examined barriers and supporting conditions for bioeconomy development using a Likert scale to measure perceived importance. The Likert scale employed is as follows: (1) Not at all important; (2) Low importance; (3) Neutral; (4) Important; and (5) Extremely important. The methodology here adopts a more practical than theoretical approach to the analysis of Likert scale responses, sidestepping traditional debates on their interpretation. Despite the responses being confined to discrete values from one to five, the mean is reported to one decimal place, helping to provide a clearer, more interpretable view of how respondents rate the importance of various barriers and supporting conditions in fostering bioeconomy development.

## Results

### Bioeconomy familiarity and information levels

The survey data provide insights into the self-rated familiarity levels of the survey’s private- and public-sector respondents and the perceived level of public information about the bioeconomy.

#### Familiarity with the bioeconomy among the public and private sectors

As a proxy to ascertain levels of familiarity with the bioeconomy in the public and private sector, survey respondents were invited to position themselves along a spectrum ranging from ‘not familiar’ (at the value 0 on the scale presented) through ‘somewhat familiar’ (at value 50) to ‘very familiar’ (at value 100). In aggregate, this produced a mean value of 63.8 across all responses received, which is within the ‘familiar’ range. Yet, it is important to note that almost half (49.6%) of the individual responses were located at the ‘high’ end of the spectrum (over 66 on the scale) and more than one-third (37.3%) in the medium range (between 33 and 66 on the scale). Only 13.1% rated their familiarity as low (under 33).

**Fig. 3:**
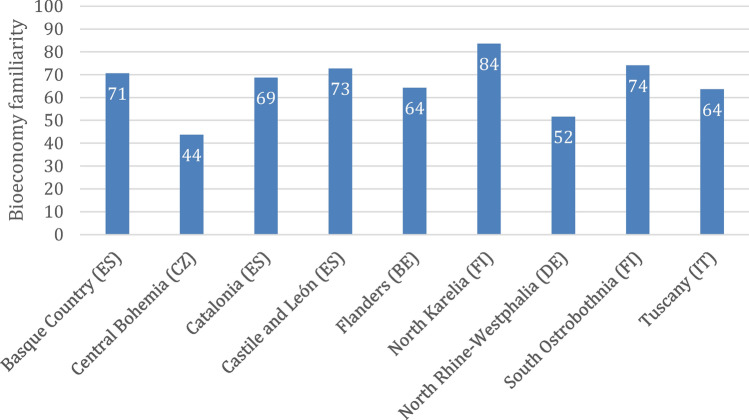
The respondents’ average familiarity with the bioeconomy in nine regions. The respondents could indicate their familiarity on a scale of 0 to 100 with 0 being not at all familiar and 100 being very familiar (Briers et al. 2024)

With reference to the mean value, the following comparison between the regions can be made (Fig. 3): bioeconomy familiarity is highest in North Karelia (83.6), followed by South Ostrobothnia (74.1), Castile and León (72.7), Basque Country (70.6), and Catalonia (68.7). It falls in the upper mid-range in Flanders (64.2), Tuscany (63.6), and North Rhine-Westphalia (51.6), and the lower mid-range in Central Bohemia (43.7). None of the regions has a mean in the low-familiarity part of the spectrum. Regarding statistical significance, familiarity in North Rhine–Westphalia and Central Bohemia is significantly lower than in the highly ranked regions such as North Karelia, South Ostrobothnia, Castile and León, the Basque Country, Catalonia, and Flanders. Tuscany, which sits in the middle of the ranking, does not differ significantly in familiarity compared to regions ranked either higher or lower.

The mean familiarity between the private and public sectors is reported at very similar levels (65 and 63, respectively); no statistically significant difference can be observed. Nor are there significant differences between different genders or age groups in these self-rated familiarity levels. However, the location of respondents—whether rural, suburban/semi-rural, or urban—does affect familiarity. The mean familiarity scores are 60 for rural areas, 67 for suburban/semi-rural areas, and 65 for urban areas. Initial analysis indicates significant differences between these groups. Further testing reveals that while the difference between rural and urban areas, and between suburban/semi-rural and urban areas, is not significant, there is a significant difference between rural and suburban/semi-rural areas.

#### General public informed on the bioeconomy

Respondents were also asked whether, in their view, the public in their region is sufficiently informed about the bioeconomy. At the aggregate level, the vast majority of respondents answered ‘the public is underinformed’ (77.7%), while only a very small proportion thought ‘the public is sufficiently informed’ (3.4%), and 7.9% selected ‘I don’t know’ (Table 1). Just over one-tenth (11.0%) of respondents did not answer this question.

**Table 1:** General public informed on the bioeconomy. Proportion of respondents (%) that find the general public is sufficiently informed on the bioeconomy, underinformed, didn’t know, or didn’t answer. Reported for all respondents together, and separately for the nine regions, the two sectors, and the three familiarity groups. Bold cells indicate those proportions that differ 20% or more from the ‘all respondents’ proportion. All figures are percentages

General public informed on the bioeconomy	All respondents	Region									Sector		Familiarity		
		Basque Country (ES)	Central Bohemia (CZ)	Catalonia (ES)	Castile and León (ES)	Flanders (BE)	North Karelia (FI)	North Rhine-Westphalia (DE)	South Ostrobothnia (FI)	Tuscany (IT)	Private sector	Public sector	Low familiarity	Medium familiarity	High familiarity
The public is sufficiently informed	3.4	0.0	0.0	2.0	2.9	5.3	**26.7**	1.8	5.4	3.8	3.3	3.5	0.0	2.0	5.3
The public is underinformed	77.7	82.9	82.2	80.6	79.4	72.4	**46.7**	82.6	67.9	80.8	77.2	78.1	77.1	74.9	80.0
I don't know	7.9	2.4	6.7	2.0	5.9	11.8	26.7	8.3	16.1	3.8	6.1	9.4	8.6	11.1	5.3
Did not answer	11.0	14.6	11.1	15.3	11.8	10.5	0.0	7.3	10.7	11.5	13.4	9.0	14.3	12.1	9.4

There is a statistically significant difference in how survey participants of different familiarity groups responded to the question, with progressively higher-familiarity (low to medium to high) respondents ascribing progressively higher information levels to the general public. No significant difference can be observed between private and public sector respondents’ answers to the question. Regional divergence in answers has been shown to be statistically significant. When comparing the proportions of different answer options of the regional subgroups to those of all respondents, it is striking that in North Karelia over a quarter of respondents think that the public is sufficiently informed, the same proportion selected ‘I don’t know’, and fewer than half think that the public is underinformed.

### Key characteristics of the bioeconomy and its development

Regarding the bioeconomy itself, respondents highlighted several important features and dynamics.

#### High-growth-potential value chains in the bioeconomy

In the next section of the questionnaire, respondents were asked to indicate up to three top value chains within the bioeconomy that they considered to have the highest growth potential in their region. The top choice—selected by over half (55.8%) of respondents—is the bioenergy value chain (Table 2). This is the most prominent answer. The next most frequent answers are wood construction, which was selected by just under a third of respondents (32.6%), green chemistry (29.8%), advanced new materials (29.2%), and food and gastronomy (28.3%). Bioplastics are mentioned by just under a quarter of respondents (24.0%), and nature-based tourism and wood-based materials and products are mentioned by nearly one in five (19.7% and 18.9%). Non-wood forest products and textiles and fashion are mentioned by 10.5% and 8.1%, respectively, and the pulp and paper value chain is seen as a high growth potential by one respondent in twenty (5.1%).

**Table 2:** Proportion of respondents (%) who indicated different value chains among the top three with the highest growth potential in their region. Reported for all respondents together, and separately for the nine regions, the two sectors and the three familiarity groups. Bold cells indicate those proportions that differ 20% or more from the ‘all respondents’ proportion. All figures are percentages

High potential value chains	All respondents	Region									Sector		Familiarity		
		Basque Country (ES)	Central Bohemia (CZ)	Catalonia (ES)	Castile and León (ES)	Flanders (BE)	North Karelia (FI)	North Rhine-Westphalia (DE)	South Ostrobothnia (FI)	Tuscany (IT)	Private sector	Public sector	Low familiarity	Medium familiarity	High familiarity
Bioenergy	55.8	56.1	55.6	70.4	66.2	**34.2**	60.0	47.7	69.6	38.5	56.1	55.6	42.9	56.8	58.5
Wood construction	32.6	**53.7**	28.9	23.5	19.1	15.8	**60.0**	52.3	39.3	**11.5**	29.3	35.4	32.9	28.6	35.5
Green chemistry	29.8	34.1	22.2	28.6	16.2	**56.6**	**0.0**	33.0	**7.1**	**50.0**	27.6	31.6	22.9	27.6	33.2
Advanced new materials	29.2	29.3	42.2	18.4	17.6	39.5	33.3	34.9	26.8	26.9	29.7	28.8	32.9	32.2	26.0
Food and gastronomy	28.3	31.7	28.9	33.7	44.1	25.0	**6.7**	**8.3**	46.4	26.9	27.2	29.2	14.3	29.6	30.9
Bioplastics	24.0	19.5	22.2	39.8	23.5	36.8	6.7	20.2	**1.8**	11.5	23.2	24.7	28.6	24.1	22.6
Nature-based tourism	19.7	9.8	28.9	17.3	26.5	2.6	**66.7**	21.1	14.3	38.5	17.9	21.2	22.9	22.1	17.0
Wood-based materials & products	18.9	14.6	8.9	15.3	11.8	10.5	13.3	35.8	26.8	15.4	18.7	19.1	20.0	18.1	19.2
Non-wood forest products	10.5	4.9	2.2	7.1	23.5	7.9	**40.0**	4.6	19.6	7.7	10.6	10.4	4.3	8.5	13.6
Textiles and fashion	8.1	4.9	0.0	8.2	4.4	18.4	6.7	4.6	1.8	**34.6**	8.1	8.0	7.1	11.1	6.0
Pulp and paper	5.1	14.6	0.0	2.0	4.4	7.9	6.7	4.6	1.8	11.5	5.7	4.5	7.1	6.0	3.8
Other	4.5	0.0	0.0	5.1	11.8	9.2	0.0	0.0	5.4	3.8	6.9	2.4	1.4	2.5	6.8

Of these, only the substantive items on ‘food and gastronomy’ and ‘non-wood forest products’ show a significant difference across familiarity levels, with higher familiarity corresponding to higher selection percentages in both cases. No significant difference was observed between private and public sector representatives. However, between the regions, there were significant differences in the rating of all the answer options. Differences in the mean proportion of respondents who selected the value chains in the individual regions, compared to the mean proportion of all respondents, are visible in Table 2. Bioenergy is selected by a comparatively low proportion of Flemish respondents. Wood construction is selected by a high proportion of respondents from the Basque Country and North Karelia, while a low proportion of respondents from Tuscany selected it. Green chemistry is considered to have a high growth potential in the respective region by a comparatively high proportion of respondents in Flanders and Tuscany and low proportion of respondents in North Karelia and South Ostrobothnia. Food and gastronomy is selected by a comparatively low proportion of respondents from North Karelia and North Rhine-Westphalia. Only a few respondents from South Ostrobothnia see potential for bioplastics in their region. A high proportion of North Karelian respondents sees high growth potential for nature-based tourism and non-wood forest products in their region. Finally, ‘Textiles and fashion’ is considered to have a comparatively high potential in Tuscany.

#### Barriers and supporting conditions for the development of the bioeconomy

When asked to rate the importance of a number of predefined barriers to the development of the bioeconomy on a Likert scale from 1 (not important at all) to 5 (very important), around one in ten respondents declined to do so for each statement (ranging from 9.9% to 10.9%). For the responses received, the following can be observed: ‘lack of co-operation among different stakeholders (e.g. policymakers, business, research)’ is rated highest with a mean value of 4.2, followed by ‘lack of a supportive policy and legislative environment well-tailored to regional needs’ with a mean value of 4.1 (i.e. both between ‘important’ and ‘very important) (Table 3). With a value of 4.0, ‘lack of profitability and market demand for bioeconomy businesses and products’ can also be considered an important barrier. ‘Lack of technical feasibility and/or barriers to innovation’ and ‘Lack of balance between different uses of forest (e.g. economic, conservation, carbon sequestration, etc.)’, both with a mean value of 3.8 are also approaching the ‘important’ marker, while ‘lack of general social acceptance’, with a mean value of 3.4, is closer to the centre mark between ‘not important’ and ‘very important’.

**Table 3:** Barriers to bioeconomy development. Respondents were asked to rate the importance of barriers to the development of the bioeconomy on a Likert scale from 1 (not important at all) to 5 (very important). Average Likert scale values are reported for all respondents together, and separately for the nine regions, the two sectors, and the three familiarity groups. Bold cells indicate those averages that differ 0.4 or more from the ‘all respondents’ proportion

Barriers (Lack of…)	All respondents	Region									Sector		Familiarity		
		Basque Country (ES)	Central Bohemia (CZ)	Catalonia (ES)	Castile and León (ES)	Flanders (BE)	North Karelia (FI)	North Rhine-Westphalia (DE)	South Ostrobothnia (FI)	Tuscany (IT)	Private sector	Public sector	Low familiarity	Medium familiarity	High familiarity
Co-operation among different stakeholders (e.g. policymakers, business, research)	4.16	4.03	4.15	4.38	4.19	4.13	4.13	4.06	3.96	4.43	4.19	4.13	3.96	4.16	4.21
Supportive policy and legislative environment well-tailored to regional needs	4.10	4.19	4.31	4.40	4.22	4.20	**3.13**	3.96	**3.50**	4.48	4.24	3.99	3.97	4.15	4.10
Profitability and market demand for bioeconomy business and products	4.00	4.11	4.00	4.15	4.12	4.14	3.67	3.86	3.92	**3.48**	3.96	4.03	3.93	4.03	3.99
Technical feasibility and/or barriers to innovation	3.80	3.68	3.85	3.82	4.13	3.65	4.00	3.63	3.88	3.83	3.76	3.83	3.72	3.89	3.75
Balance between different uses of forest (e.g. economic, conservation, carbon sequestration, etc.)	3.74	3.59	4.00	3.97	3.78	3.43	3.53	3.76	3.67	3.74	3.75	3.73	3.83	3.79	3.69
General social acceptance	3.42	3.49	**3.82**	3.66	3.43	3.48	**2.80**	3.37	3.02	**3.00**	3.41	3.43	3.58	3.41	3.39

Interestingly, there are no significant differences between groups with different levels of familiarity. A significant difference between the private and public sector was found only for the assessment of a lack of a supportive policy and legislative environment well-tailored to regional needs (with the mean of private sector respondents being slightly higher than that of public sector respondents), but not for the other predefined barriers.

By contrast, the regional breakdown of the data showed significant differences for all the barriers apart from ‘lack of profitability and market demand for bioeconomy businesses and products.’ For ‘lack of co-operation among different stakeholders’, there is a statistically significant overall difference among the regions, but subsequent pairwise comparisons do not reveal significant differences between specific regions.^3^ With regard to ‘lack of supportive policy and legislative environment well-tailored to regional needs’, there is a significant difference for North Karelia and South Ostrobothnia compared to all other regions except between each other and with North Rhine-Westphalia, as well as between North Rhine-Westphalia and Catalonia. Whenever there is a significant difference between the pairs of regions, respondents of North Karelia, South Ostrobothnia and North Rhine-Westphalia considered the barrier less important than the respondents in the other region in the pair. For’lack of technical feasibility and/or barriers to innovation’, the only significant differences observed are that Castile and León respondents consider it more important than respondents from Flanders and from North Rhine-Westphalia. With regard to ‘lack of balance between different uses of forest’, Flemish respondents considered it less important than Catalan respondents. ‘Lack of general social acceptance’ is considered less important by respondents from South Ostrobothnia than by those from Catalonia and Central Bohemia.

Asked about the conditions supporting the development of the bioeconomy, the rate of declined responses also hovers around one in ten, though it is slightly lower than for the barriers (ranging from 9.4 to 10.3%). The top two responses are firmly on the ‘important’ to ‘very important’ side of the scale: ‘Public/private investment in innovation’ has a 4.4, while ‘availability of scientific information for a better-informed public and policymakers’ has a mean value of 4.3 (Table 4). Although placed, in their aggregate, between the central marker and the ‘important’ marker of the scale, the remaining three options all tend towards ‘important’, too: ‘Adequate regulation to overcome possible negative impacts on ecosystems and local communities’ has a mean of 3.9, ‘public procurement programmes, to stimulate demand’ has a mean of 3.8, and ‘performance-based payments for carbon sequestration’ has a mean of 3.7.

**Table 4:** Supporting conditions for bioeconomy development. Respondents were asked to rate the importance of supporting conditions for the development of the bioeconomy on a Likert scale from 1 (not important at all) to 5 (very important). Average Likert scale values are reported for all respondents together, and separately for the nine regions, the two sectors and the three familiarity groups. Bold cells indicate those averages that differ 0.4 or more from the ‘all respondents’ proportion

Supporting conditions	All respondents	Region									Sector		Familiarity		
		Basque Country (ES)	Central Bohemia (CZ)	Catalonia (ES)	Castile and León (ES)	Flanders (BE)	North Karelia (FI)	North Rhine-Westphalia (DE)	South Ostrobothnia (FI)	Tuscany (IT)	Private sector	Public sector	Low familiarity	Medium familiarity	High familiarity
Public/private investment in innovation	4.42	4.43	4.10	4.63	4.44	4.33	4.67	4.30	4.42	4.68	4.43	4.41	4.25	4.34	4.51
Scientific information for better-informed public and policymakers	4.25	4.33	4.31	4.28	4.28	4.03	4.33	4.27	4.26	4.41	4.26	4.25	4.27	4.13	4.34
Adequate regulation to overcome possible negative impacts on ecosystems and local communities	3.93	4.08	3.79	**4.36**	3.95	4.01	4.20	3.61	**3.52**	4.22	3.94	3.93	3.83	3.96	3.94
Public procurement programmes, to stimulate demand	3.81	**4.22**	**3.41**	4.03	3.85	3.80	4.20	3.46	4.10	3.61	3.77	3.84	3.64	3.67	3.95
Performance-based payments for carbon sequestration	3.67	3.59	3.32	3.77	3.67	3.54	3.87	3.67	3.94	3.68	3.69	3.66	3.63	3.61	3.72

With regard to these supporting conditions, in contrast to the barriers discussed above, bioeconomy familiarity levels of respondents did make a difference for their estimation of the importance of three out of the five items: availability of information to inform the public and policymakers, investment in innovation, and public procurement programmes to stimulate demand. Public procurement programmes were rated significantly more important by respondents of the highest familiarity group in comparison with the two other familiarity groups. Investment in innovation was rated significantly more important by respondents in the high-familiarity group than by those in the low-familiarity group. With regard to availability of information to inform the public and policymakers, the high-familiarity group rated it significantly more important than the medium-familiarity group, and no significant differences were observed compared with the low-familiarity group.

There were no significant differences observed between respondents from different sectors on any of the items. However, respondents from different regions had different estimations of the importance of available investment, relevant regulation, and public procurement programmes in their region. With regard to investment in innovation, Catalan respondents rated it significantly more important than respondents from North Rhine-Westphalia, Flanders, and Central Bohemia, and Tuscany respondents rated it more important than respondents from Central Bohemia. ‘Adequate regulation to overcome possible negative impacts on ecosystems and local communities’ was rated as significantly more important by respondents from Catalonia compared to Central Bohemia, North Rhine-Westphalia, and South Ostrobothnia; it was also rated of higher importance by Tuscan respondents than South Ostrobothnia respondents. The importance of ‘public procurement programmes to stimulate demand’ was rated significantly less important by Central Bohemia and North Rhine-Westphalia respondents, compared to North Karelia, South Ostrobothnia, Catalonia, and Basque respondents.

### Willingness and responsibility to develop the bioeconomy

Finally, respondents were also asked several questions to gauge their willingness to develop the bioeconomy and whether they perceive the public or private sector, or both, to be responsible for doing so.

Willingness to develop the bioeconomy was assessed both in terms of abstract intentions and in terms of concrete past actions, such as investments for private sector respondents and regulatory efforts for public sector respondents. It is important to note that the non-response rate was rather high for these questions: just over one-third among private sector respondents (35.0% and 33.7%, respectively) and just under that value for public sector respondents (29.5% and 31.9%).

Respondents gave an average score of 74.9 (on a slider scale of 0–100) when asked about their willingness to develop the bioeconomy (Table 5). Therefore, the respondents seem rather willing to develop the bioeconomy. When asked about their past actions to develop the bioeconomy, nearly two-thirds of respondents (62.8%) indicated that they had been involved in such activities.

Subsequent statistical analysis of the reported willingness showed that the high-familiarity group had a significantly higher willingness to develop the bioeconomy compared to the medium- and low-familiarity groups.

Looking at the two groups of public versus private sector representatives separately, private sector respondents were significantly more willing to develop the bioeconomy than public sector actors. The average willingness scores of private and public sector respondents are 77.5 and 72.8, respectively (Table 5).

**Table 5:** Willingness and concrete past actions for bioeconomy development. The willingness to develop the bioeconomy is reported as a mean value based on the scores given on a slider scale of 0–100. The concrete past bioeconomy development actions are reported as the proportion (%) of respondents who indicated that they had been involved in such activities. The values are reported for all respondents together, and separately for the nine regions, the two sectors, and the three familiarity groups. For the willingness variable, Bold cells indicate those average values that differ by more than 10 from the ‘all respondents’ average. In the case of the past bioeconomy development actions, Bold cells indicate those proportions that differ 20% or more from the ‘all respondents’ proportion

	All respondents	Region									Sector		Familiarity		
		Basque Country (ES)	Central Bohemia (CZ)	Catalonia (ES)	Castile and León (ES)	Flanders (BE)	North Karelia (FI)	North Rhine-Westphalia (DE)	South Ostrobothnia (FI)	Tuscany (IT)	Private sector	Public sector	Low familiarity	Medium familiarity	High familiarity
Willingness to develop the bioeconomy (mean)	74.9	77.5	**56.2**	80.9	82.4	73.8	82.7	64.9	76.5	83.8	77.5	72.8	**61.0**	67.5	83.0
Concrete past bioeconomy development actions (%)	62.8	**87.1**	**33.3**	50.0	**85.7**	72.6	60.0	47.8	46.8	81.0	66.9	59.6	**31.0**	51.5	77.6

In a regional comparison, too, statistically significant differences can be observed. Central Bohemian respondents report a significantly lower willingness to develop the bioeconomy than the respondents in North Karelia, Catalonia, South Ostrobothnia, the Basque Country, Tuscany, and Castile and León. Respondents from North Rhine-Westphalia report a significantly lower willingness than respondents from Castile and León.

With regard to past actions to develop the bioeconomy, there is a significant difference between familiarity groups. Looking at the data, a higher proportion of respondents in the high-familiarity group (77.6%) indicated that they had been involved in such activities, compared to the medium (51.5%) and low (31.0%) familiarity groups (Table 5). Here, it is interesting to note that even among the lowest-familiarity group, nearly a third of respondents (31.0%) had previously undertaken an activity to develop the bioeconomy.

The difference between private and public sector respondents is not significant, while there is a significant difference between regions. Looking at the proportions of respondents within the regions, a comparatively high proportion of respondents from the Basque Country and Castile and León had previously been involved in activities to develop the bioeconomy, while only a low proportion of Central Bohemian respondents had.

The next question aimed at finding out where responsibility for developing the bioeconomy lies. The statements respondents were presented with were: Who is responsible for ensuring the positive environmental and social impacts of the bioeconomy? Who should invest in research, development, and innovation? Who is responsible for communicating and promoting the bioeconomy among the public? It should be noted that a large proportion, more than one in five respondents, declined to answer this question for all three statements (21.9%, 23.4%, and 21.3%, respectively).

**Fig. 4:**
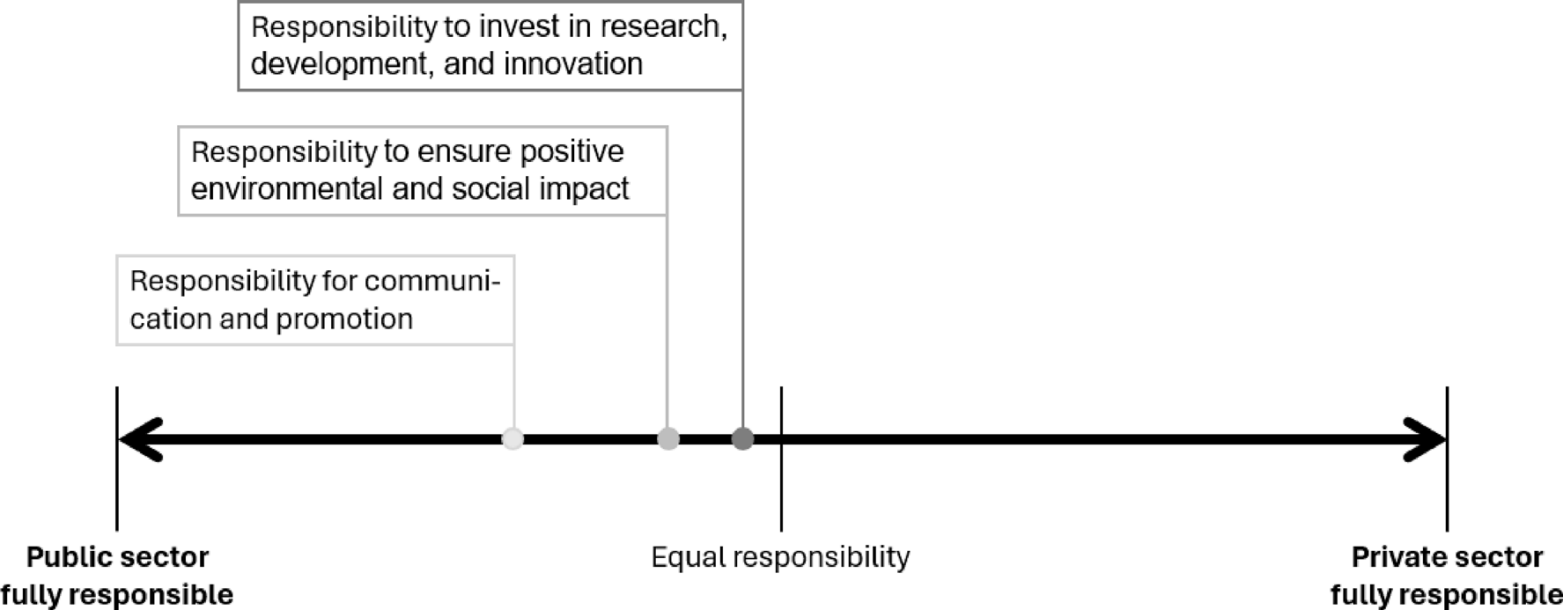
Responsibilities for three tasks for moving the bioeconomy forward, along a spectrum from full public sector responsibility to full private sector responsibility

With all mean values below the central (i.e. equal responsibility) marker, the results show that both groups consider each of these responsibilities to rest more with the public sector than the private sector (Fig. 4, Table 6). At a mean value of 29.8 (29.7 among private sector actors and 29.8 among public sector actors), this is particularly true for the responsibility for communication and promotion. The result is less pronounced for the responsibility to ensure positive environmental and social impacts (here, the aggregate mean is 41.5, while the one for the private sector is 40.7 and that of the public sector is 42.1) and even less pronounced, i.e. approaching the centre value (aggregate mean: 47.2, consisting of private sector 48.9 and public sector 45.8) for the responsibility to invest in the bioeconomy. When looking at the aggregate data, the distribution of values for all three tasks are significantly different from the centre value (50), meaning that the public sector is considered to be more responsible for all three activities promoting the bioeconomy.

**Table 6:** Responsibilities for three tasks for moving the bioeconomy forward. Reported as mean values based on the scores given on a continuous slider scale of 0–100 along a spectrum from full public sector responsibility at value 0, through equal responsibility of both sectors at the centre marker 50, to full private sector responsibility at value 100. The values are reported for all respondents together, and separately for the nine regions, the two sectors, and the three familiarity groups. Bold cells indicate those average values that differ by more than 5 from the ‘all respondents’ averages

Tasks in moving the bioeconomy forward (Who is responsible for…)	All respondents	Region									Sector		Familiarity		
		Basque Country (ES)	Central Bohemia (CZ)	Catalonia (ES)	Castile and León (ES)	Flanders (BE)	North Karelia (FI)	North Rhine-Westphalia (DE)	South Ostrobothnia (FI)	Tuscany (IT)	Private sector	Public sector	Low familiarity	Medium familiarity	High familiarity
Ensuring the positive environmental and social impacts of bioeconomy?	41.5	44.1	40.5	43.5	41.0	39.0	43.5	40.1	45.1	38.3	40.7	42.1	42.6	42.8	40.2
Investing in research, development, and innovation?	47.2	50.5	**42.1**	51.4	48.2	47.9	49.3	43.5	47.4	45.5	48.9	45.8	45.8	47.0	47.7
Communicating and promoting bioeconomy among the public?	29.8	**23.2**	26.4	30.0	30.1	**35.4**	26.7	28.8	32.8	27.0	29.7	29.8	25.8	33.4	27.9

With regard to the different familiarity groups, there is a significant difference between the groups for the responsibility for communication and promotion, there is no significant difference for the other two tasks. With regard to communication and promotion, subsequent statistical analysis shows that the low- and high-familiarity groups attribute a significantly higher responsibility to the public sector, than the medium-familiarity group. For none of the three tasks is there a significant difference between how public and private sector respondents or respondents from different regions perceive the responsibilities. This means that across sectors and regions, respondents agree that the responsibility to develop the bioeconomy rests more with the public sector than the private sector.

## Discussion

Looking at the aggregate of responses to specific questions, as well as responses across various respondent groups by familiarity, sector, and regional affiliation, two main themes emerge from the data as pivotal considerations in assessing the trajectory or pathways of bioeconomy development. The first is the overarching need to consider regional specificity, as evidenced in all parts of the analysed survey. The second is the identification of specifics regarding which actors should foster the bioeconomy transition and how. These insights are summarised below, with the aim of informing recommendations to regional public sector institutions, governments, and administrations, which are ideally positioned to take ownership of and drive bioeconomy transition processes.

### Regional specificities

Although much of the literature treats bioeconomy locations as coincidental, as pointed out by Briers et al. (2024), it would be naïve to assume that on-the-ground conditions such as familiarity with the bioeconomy and availability of bio-based resources do not influence bioeconomy practice.

Regarding familiarity with the bioeconomy, the data presented in the study show that some regional differences coincide with the comparative familiarity levels of those regions (Fig. 3). Nevertheless, the regional specificity of external circumstances, such as availability of bio-based resources and practices, also plays a role. These take the form of long-standing local value chains and traditional cultural practices, as well as emphases on promising fields for present and future economic activity. All these foci also impact perceptions, the attribution of responsibilities, and assessments of conditions for moving the bioeconomy forward. Hence, a ‘one-size-fits-all’ approach does not work, and the bioeconomy is regionally specific, or—drawing on policy terminology—‘place-based’ as also found by Morales (2022).

The results demonstrate how regional conditions affect the respondents’ perceptions of the bioeconomy and its dynamics. For instance, regions with an established focus on particular value chains, such as Tuscany in fashion and textiles, or Tuscany and Flanders in chemistry, more strongly select the corresponding bio-based value chains as having high potential than other regions. Wood construction is selected by a comparatively high proportion of respondents from the Basque Country and North Karelia. Respondents in the latter also see high growth potential for non-wood forest products in their region.

Regarding the identification of promising fields for economic activity that are more recent or future-oriented, North Karelian respondents’ frequent selection of nature-based tourism as a value chain of high growth potential (Table 2) is a case in point, as Ana (2017) found for other European regions.

In five out of the nine regions, bioenergy is selected as the top value chain with the highest growth potential (Table 2). This may come as a surprise considering the emphasis respondents place on sustainability and other environmental benefits (Briers et al. 2024). This presents a challenge, as the utilisation of resources for fuel contradicts the principles of multiple use and circularity inherent in other bioeconomy value chains (Lokesh et al. 2018; Lewandowski et al. 2019; Sherwood 2020; Muscat et al. 2021). It may be that the high rating of bioenergy as a high-growth-potential value chain resulted, in part, from the skyrocketing energy prices during the period that the survey was open to respondents (autumn 2021 to winter 2022) and which was also discussed in scholarly literature (Winchester and Reilly 2015; Winchester and Ledvina 2017).

At the same time, this discrepancy between the emphasis on sustainability and the prioritisation of bioenergy highlights an ongoing tension within the evolving bioeconomy narrative. Such contradictions could stem from an overly optimistic or insufficiently critical interpretation of the bioeconomy’s potential, reflecting a need for a more balanced discourse. Left unaddressed, these inconsistencies may risk undermining the credibility of the bioeconomy framework itself, as previous studies have warned (Birch 2017; Eversberg et al. 2022; Briers et al. 2024).

Two out of the four regions where bioenergy is not the top selected value chain with high growth potential are Flanders and North Rhine-Westphalia (Table 2). Both are regions with a limited biomass availability per capita, which can be a reason for increased awareness of the effective and sustainable use of biomass. On the opposite side, Catalonia and Castile and León are the regions where bioenergy was selected as a sector with high growth potential by the highest share of respondents. Both are Mediterranean regions coping with the challenge of unmanaged forests, stemming from issues such as fragmented private ownership and low value of biomass (Martinez de Arano et al. 2018). This has resulted in landscapes increasingly dominated by continuous, high-density, young forests, which pose an extreme wildfire risk. In such regions, using biomass for energy can be a valuable strategy to ensure increased forest management despite the low value of biomass (Fernandes 2013; Madrigal et al. 2017).

Regarding the rating of barriers and supporting conditions, variation can also be observed between the studied regions. While generally rated high in importance as a barrier to the bioeconomy across regions, lack of supportive policy and legislative environments well-tailored to regional needs is scored as less of a barrier in North Rhine-Westphalia and in the Finnish regions, North Karelia and South Ostrobothnia, compared to the other regions (Table 3). Interestingly, the Finnish regions have a long track-record of bioeconomy activities, and it plays an important role in the regions’ GDP and employment (also discussed in Simola et al. 2010; Refsgaard et al. 2021). Therefore, policymakers are likely to have given more importance to developing a supportive environment for the bioeconomy. Both this particular case and the speculation about general dynamics offered here should be investigated further.

Regarding ‘public procurement programmes, to stimulate demand’, there are regional differences observable in that Catalan respondents, together with those from the Finnish regions and the Basque Country, rated their importance higher than those from Central Bohemia and North Rhine-Westphalia (Table 4). Simultaneously, Catalan respondents also consider investment in innovation a significantly more important supporting condition than respondents from North Rhine-Westphalia, Flanders, and Central Bohemia. In addition, Catalan respondents emphasised ‘adequate regulation to overcome possible negative impacts on ecosystems…’ as more important than respondents from North Rhine-Westphalia, Central Bohemia, and South Ostrobothnia. While it is beyond the scope of this study to fully interpret these differences—e.g. by contextualising the findings with further information and analysis of regional governmental and governance approaches—further research in this field is needed to inform successful place-based bioeconomy development. This is particularly true as interventions such as the creation of a supportive policy and legislative environment, public procurement, and adequate regulation fall firmly into the remit of the public sector, whose responsibility for the development of the bioeconomy is discussed below.

The current policy context in which regions operate is another factor influencing regionally specific perceptions of the bioeconomy, as demonstrated by Briers et al. (2024) and exemplified by the current paper, that shows that respondents from Castile and León consistently selected bioenergy as the value chain with the highest growth potential (Table 2). This is consistent with the strategic direction set by the Castile and León Bioenergy Plan (Government of Castile and León 2011), which demonstrates the influence of long-term strategic planning and how it permeates the priorities of local stakeholders. Similarly, in most of the regions discussed here, some alignment is observable between the priority value chains identified by survey respondents and regulatory activities in their territory (Tables S1 and S2). Where this is not the case, further research should explore to what extent this is due to diverging terminology or a substantial misalignment of policy and practical priority.

More generally, regional bioeconomy policies, strategies, and implementation plans are sometimes the result of bottom-up alignment with national counterparts, and sometimes a top-down necessity in the implementation of a national government’s strategy according to the subsidiarity principle. The latter is the case in North Karelia, which was the first region in Finland to create a regional implementation plan for the National Bioeconomy Strategy (Regional Council of North Karelia 2023). In the case of the Czech Republic, a centralised country with regions having limited autonomy and capacity, the absence of a national bioeconomy strategy is seen as a key reason for the inexistence of regional bioeconomy strategies (Rinn et al. 2023). Here, the national level is seen as the crucial intermediate link between regional and EU levels, and the state is expected to adopt a national bioeconomy strategy to create a model for individual regions, such as Central Bohemia. As long as the Czech national strategy is missing, the influence and impact of the EU strategy at the regional level are expected to be small.

On the other hand, there are regions with more autonomy and capacity among the analysed ones, such as the Spanish regions (Catalonia, Basque Country, Castile and León), North Rhine-Westphalia, and Flanders. For such regions, regional bioeconomy policies and strategies are generally less influenced by the undertakings of the national government. For example, Flanders published a first regional bioeconomy strategy in 2013, while at the national level in Belgium there is none to date, as the development and implementation of bioeconomy policies are managed at the regional level (Government of Flanders 2013; Van Kerckhove et al. 2023). Catalonia and Castile and León also mention the EU bioeconomy strategy as a reference and inspiration for regional bioeconomy-related policies and strategies, such as the Catalan Bioeconomy Strategy (Government of Catalonia 2021), and the Sectoral Habitat Plan from Castile and León (Government of Castile and León 2022). This exemplifies how the policy context of a region, including the (de)centralisation of decision-making, affects the policies and strategies available at the regional level, as well as their content, and the influence of the European Bioeconomy Strategy at the regional level.

### Who and how to foster the bioeconomy transition

Even aside from regional specificities, the development of the bioeconomy is a complex process relying on different pathways, dynamics, and actors—these may be based in research or applied contexts (Hogarth and Salter 2010; De Besi and McCormick 2015), economic or social endeavours, etc., or in either of the two sectors discussed in this study.

Respondents from the public and the private sector both stated high levels of concrete past bioeconomy development actions (Table 5). Also, resonating with previous research on defining elements of the bioeconomy (Briers et al. 2024), the findings presented in this paper underscore that representatives from the public and the private sector generally share broad understandings of the bioeconomy’s dynamics and well-placed actors. These findings can be considered a very strong foundation for the further co-development of the bioeconomy by the two groups.

However, the results presented here also highlight some differences—particularly regarding the sectors’ roles in its development—that need to be considered in applied contexts to avoid risks to its effectiveness. Both the reassuring similarities and potentially risky divergences are discussed in the following paragraphs.

Despite the similarity in past involvement in the bioeconomy, there is a significant difference between the two sectors. Private sector respondents show a somewhat more pronounced willingness than those from the public sector to engage proactively in the development of the bioeconomy (Table 5), which has also been found by Kircher et al. (2018). Conversely, lower willingness of public sector actors may not only be viewed as a potential risk in view of the high hopes attached to the public sector’s regulatory function by analysts as outlined in the introduction (Thran and Moesenfechtel 2022). The survey findings show that respondents from both sectors also assign the public sector more responsibility in developing the bioeconomy in all areas offered for assessment: ensuring positive environmental and social impacts; investing in research, development, and innovation; and communicating and promoting the bioeconomy among the general public (Table 6).

Incidentally, both sector groups agree not only in their attribution of responsibility for communication and promotion to the public sector (Table 6), but also on their perception that lack of general social acceptance is a somewhat important barrier to bioeconomy development and that the general public is underinformed about the bioeconomy (Table 1). The latter is in contrast with their self-perception of being familiar with the bioeconomy. This can indicate the need for more targeted and effective communication strategies to raise public awareness and understanding of the bioeconomy, as knowledge and familiarity are identified in the literature and discussed above as important preconditions for public buy-in and support of bioeconomy activity and development (Stern et al. 2018; Woźniak et al. 2020). Initiatives such as educational campaigns, community engagement programmes, and the integration of bioeconomy topics into educational curricula could help bridge this apparent knowledge divide (Paris et al. 2023; Pink et al. 2024). Additionally, fostering collaborations between governments (on regional and local levels), industry, and the media may enhance visibility and public information, creating a more supportive environment for bioeconomic development, as also discussed by Refsgaard et al. (2021).

However, while their agreement on responsibility for bioeconomy development is reassuring, two important divergences need to be highlighted. The first is between the public sector’s stated willingness and its commonly perceived responsibility: while this divergence is not glaring, it may nevertheless pose risks to the effective development of the bioeconomy that should not be overlooked—especially by public sector actors who might otherwise fail to fulfil the role that both they themselves and the private sector assign to them. Further conversation, collaboration, precise definition of respective roles, and development of additional public sector practice may be needed. Third parties, such as research and policy organisations, may be of use in providing platforms to facilitate these processes and to identify and inform regulatory needs and opportunities.

The second important divergence that emerges from the survey is that when it comes to identifying important potential barriers to the successful development of the bioeconomy, private sector respondents express a significantly higher concern about the lack of a conducive policy and regulatory environment (also observed by Bugge et al. 2016; Dupont-Inglis and Borg 2018; Dietz et al. 2023). Conversely, the public sector expresses a lower concern for this potential barrier. Simultaneously, the public sector is uniquely placed to prevent it from manifesting (because it oversees creating policy and regulation) and perceived by all actors to be prominently responsible for the development of the bioeconomy. This means that there is a danger that the public sector might overlook the importance of this issue as well as misjudge its own role in creating or preventing it. To ensure that a conducive policy and regulatory context is created, public sector representatives will do well to take into account the views and concerns of the private sector. This will do justice to high-level economic and environmental risks prioritised by the former and ‘on-the-ground' risks and benefits affecting individuals or companies as prioritised by the latter (Briers et al. 2024). In addition, further research may elicit the nature of sector-specific practical and theoretical understandings as well as any conflicting interests or dynamics, to facilitate constructive collaboration between both sector groups and to mediate interests.

## Conclusion

This paper examined the perceptions of public and private sector stakeholders across nine European regions. Key findings include the regional specificity of the bioeconomy and its perceptions, and largely similar perceptions of the public and private sectors, although some important differences in perceptions must be taken into account for future bioeconomy development pathways. Specifically, the following takeaways regarding bioeconomy development should be noted: (i) both sectors assigned more responsibility to the public sector; (ii) the public and the private sector demonstrated high willingness; (iii) respondents more familiar with the bioeconomy show a higher willingness; (iv) access to investment and scientific knowledge are the most prominent supporting conditions; (v) limited cooperation among stakeholders and an inadequate policy and legislative environment are the primary barriers.

Further research could deepen understanding of optimal bioeconomy development by exploring governance roles, key stakeholder information flows, and critical structures or mechanisms (e.g. funding, research, intellectual property, and public–private partnerships, etc.) needed to sustain momentum and success. In parallel, comparative work on how the bioeconomy is defined—both formally in policy documents and informally by practitioners—would clarify whether observed differences across regions stem from substantive realities or merely from semantic variation, and whether regulatory activities correspond to relevant actors’ priority value chains.

Various types of complexity of the bioeconomy also warrant further study. In-depth regional studies are needed to shift from treating place as incidental to recognising place-based factors as decisive for meaningful analysis of each region’s potential, status quo, barriers, and enabling conditions. How to reconcile competing value chains or uses of biomaterials and competing sectoral interests, and how to ensure equitable access to the benefits produced by the bioeconomy are questions to which research has a role in contributing answers.

Recommendations for practical actions and approaches can also be distilled from the analysis presented here. Most obviously, potential barriers (e.g. lack of stakeholder collaboration and an unhelpful policy and legislative environment) need to be addressed, and important supporting conditions (e.g. access to investment and scientific knowledge) maximised. The public sector should take on the responsibility that it assigned as per consensus.

The divergence of practice from attitudes regarding responsibilities and willingness for bioeconomy development seems to necessitate further conversation, collaboration, definition of respective roles, and additional public sector engagement. Third parties, such as research and policy organisations, may be of use in providing platforms to facilitate these processes, and to identify and inform regulatory opportunities in order to align stakeholder perspectives.

Regional-level actors should be empowered to shape their bioeconomy based on their unique assets and opportunities. Where there are similarities of place-based features, or of difficulties to overcome, inter-regional knowledge sharing can foster mutual learning and problem-solving.

Finally, all actors involved in the bioeconomy should take care to not pursue the bioeconomy as yet another (extractive and exploitative) economic endeavour but to do justice to the sustainability aspirations associated with the term. This is important to the actors, as represented in the present study, themselves, as well as crucial in retaining and bolstering the general public’s trust in and support for the bioeconomy.

## Supplementary Information

Below is the link to the electronic supplementary material.

Supplementary file1 (PDF 425 kb)

## Footnotes

1 During the preparation of this paragraph, the authors used DeepSeek's DeepSeek-V3 language model to synthesise the responses received on the corresponding open-ended question in the survey in eight different languages. This was intended to address the complexities created by potentially diverging nuances of sector definition and meaning of terminology in different languages, and the language use to convey similar meanings. It was deemed that the technology could, thus, produce the best possible approximation for attributing value chain backgrounds of respondents. To sense check the resulting interpretation presented here, the authors reviewed the steps undertaken by DeepSeek and found that they were appropriate for creating an accurate outcome.

2 It is important to note that the term "sectors," as used in the survey questionnaire, has been replaced with "value chains" throughout this paper to prevent confusion with the terms "public sector" and "private sector." While some sectors, such as forestry and agriculture, encompass multiple distinct value chains, this paper does not further differentiate between them in its terminology.

3 This phenomenon is not uncommon in statistical analysis and can be attributed to several factors, a discussion of which would go far beyond the space available here. Readers may familiarise themselves with this topic by reading up on the relevant literature.
