# Supplementary material for: Pathways to bioeconomy development: A multi-regional perspective from Europe
Source: Ambio. 2025 Dec 3;55(6):1264–92. doi: 10.1007/s13280-025-02297-4 (PMC13125476; doi:10.1007/s13280-025-02297-4)
Supplement: Supplementary file 1 — Supplementary file1 (PDF 425 kb) [file 13280_2025_2297_MOESM1_ESM.pdf]

AMBIO: Supplementary Information

Article title: Pathways to bioeconomy development: A multi-regional perspective from Europe

Authors: Siebe Briers, Anne Ackermann, Ivana Živojinović, Stefanie Linser, Radek Rinn, Inazio Martinez de Arano, Johanna Klapper, Venla Wallius, Melanie Amato Kriján, Leire Barañano Orbe, Miriam Gonzalez Dominguez, Sari Koivula, Gudrun Van Langenhove, Stefanie Wieland

Table of Content

- Supplementary material 1

  Table S1. Adoption status (as of 2022) of bioeconomy-related strategies in the nine studied regions and the value chains and areas of activity addressed

- Supplementary Material 2

  Survey used in the study

Supplementary Material 1

Table S1. Adoption status (as of 2022) of bioeconomy-related strategies in the nine studied regions and the value chains and areas of activity addressed. Value chains are ranked alphabetically.

**Region (country)**	**National bioeconomy strategy**	**Value chains and areas of activity addressed**	**Regional bioeconomy-related strategies and frameworks**	**Value chains and areas of activity addressed**
Basque Country (ES)	Spanish Bioeconomy Strategy: Horizon 2030 (Government of Spain, 2016)	• Agriculture<br>• Aquatic, marine, algae<br>• Bioenergy and biofuels<br>• Biotechnology<br>• Blue growth<br>• Biomaterials<br>• Food<br>• Forestry<br>• Waste	Circular Economy Strategy of the Basque Country 2030 (Government of Basque Country, 2019); Circular Economy and Bioeconomy Plan 2024 (Government of Basque Country, 2021)	• Agriculture<br>• Agri-food<br>• Construction<br>• Forestry<br>• Innovation<br>• Marine<br>• Re-use<br>• Research<br>• Sustainable materials<br>• Waste
Catalonia (ES)	*ibid.*	*ibid.*	Catalan Bioeconomy Strategy 2030 (Government of Catalonia, 2021)	• Agriculture<br>• Agri-food<br>• Financial<br>• Forestry<br>• Maritime<br>• Research
Castile & León (ES)	*ibid.*	*ibid.*	Castile and León Circular Economy Strategy 2021-2030 (Government of Castile and León, 2021); Castile and León Bioenergy Plan (Government of Castile and León, 2011); Sectoral Habitat Plan from Castile and León (Government of Castile and León, 2022)	• Agriculture<br>• Agri-food<br>• Construction<br>• Energy<br>• Industry<br>• Infrastructure<br>• Manufacturing<br>• Recycling<br>• Research<br>• Textile industry<br>• Tourism<br>• Waste<br>• Water
North Karelia (FI)	Finnish Bioeconomy Strategy (Government of Finland, 2014; Government of Finland, 2022)	• Agri-food<br>• Forestry<br>• Forest-based industries<br>• Water<br>• Biorefinery	North Karelian Smart Forest Bioeconomy Strategy (Regional Council of North Karelia, 2018)	• Bio-based products<br>• Bioenergy<br>• Biorefinery<br>• Forestry<br>• Forest technology<br>• Forest information<br>• Wood Construction<br>• Research<br>• Transport
South Ostrobothnia (FI)	*ibid.*	*ibid.*	Regional Strategy of South Ostrobothnia (Regional Council of South Ostrobothnia, 2021)<br>Smart Specialisation Strategy 2021-2027 (Regional Council of South Ostrobothnia, 2022)	• Bioenergy<br>• Biotechnology<br>• Construction<br>• Education<br>• Food industry<br>• Recreation<br>• Tourism<br>• Transport

Central Bohemia (CZ)	No dedicated national strategy	-	Circular Economy Action Plan for Central Bohemia 2022+ (Central Bohemian Innovation Center, 2022); Circular Scan of the Central Bohemia Region (Central Bohemian Innovation Center, 2021)	• Agriculture<br>• Car industry<br>• Fishery<br>• Food<br>• Forestry<br>• Waste<br>• Water
Flanders (BE)	No dedicated national strategy	-	Bioeconomy in Flanders: Vision & Strategy 2030 (Government of Flanders, 2013)	• Aquaculture<br>• Agriculture<br>• Biotechnology<br>• Biochemicals<br>• Bioenergy<br>• Fishery<br>• Food<br>• Forestry<br>• Industry<br>• Logistics<br>• Marine<br>• Research<br>• Waste
North Rhine-Westphalia (DE)	German National Bioeconomy Strategy (Government of Germany, 2020)	• Agriculture<br>• Industry<br>• Biogenic materials<br>• Construction<br>• Energy<br>• Food<br>• Fisheries<br>• Forestry<br>• Research	Bioeconomy in NRW (Exzellenz Cluster NRW, 2013); strategy drafting ongoing	• Agriculture<br>• Biotechnology<br>• Environmental management<br>• Fisheries/ aquaponics<br>• Food/nutrition<br>• Forestry<br>• Science<br>• Waste
Tuscany (IT)	BIT II – Italian Bioeconomy Strategy (Government of Italy, 2019)	• Agriculture<br>• Bio-based industry<br>• Food industry<br>• Forestry<br>• Marine and maritime industries	Smart Specialisation Strategy (S3) Tuscany 2021-27 (Government of Tuscany, 2022)	• Agriculture<br>• Biotechnology<br>• Energy<br>• Food<br>• Forestry<br>• Healthcare<br>• Mobility<br>• Research<br>• Space economy<br>• Tourism<br>• Waste

BIOREGIONS
Forest bioeconomy in action

Bioeconomy Perceptions Survey

Welcome!

You are being invited to participate in this survey by (REGION), with support from the Bioregions Facility at the European Forest Institute.

The purpose of this study is to learn more about how different groups within government and industry perceive the bioeconomy, its benefits, and its challenges.

The survey is divided into two parts and will take approximately 15 minutes to complete. In part I we will ask your opinion about the concept of bioeconomy in general, its benefits and risks. In part II we will ask your opinion about different aspects of bioeconomy specifically in (REGION). At the end of the survey, we will give you the opportunity for further reflections.

Your participation in this study is completely voluntary, and you can exit the survey at any time if you do not wish to continue. All results are collected anonymously and your contact information will not be retained.

You can return to this link to edit your answers or complete the survey at any time.

By clicking 'OK' below you indicate that you have read and understood this consent form and agree to participate in this study.

BIOREGIONS
Forest bioeconomy in action

Bioeconomy Perceptions Survey
Demographic questions

* 1. In which field do you work?

◯ Industry (or related private sector)

◯ Government (or related public sector)

◯ Other (please specify)

2. What is the name of your sector / government department?

3. What is your job position?

* 4. Gender

◯ Female

◯ Male

◯ Other

◯ Prefer not to say

* 5. Age

◯ 18-24

◯ 25-34

◯ 35-44

◯ 45-54

◯ 55-64

◯ 65+

* 6. Location

◯ I live in (REGION)

◯ I work in (REGION)

◯ Both

* 7. Which of the following best describes the area where you live?

◯ Urban

◯ Rural

◯ Suburban or semi-rural

BIOREGIONS
Forest bioeconomy in action

Bioeconomy Perceptions Survey

Part I: Bioeconomy as a concept, benefits and risks

There are several definitions of bioeconomy. The definition used in this survey is "an economy that relies on the production and utilisation of renewable biological resources, including those of forest origin, to produce materials, energy, products, and services across all economic sectors."

* 8. How familiar are you with the bioeconomy?

Note that you can locate the slider at any point of the scale.

Not familiar — Somewhat familiar — Very familiar

9. Which of the following concepts do you understand to be part of the bioeconomy?

- ☐ Nature-based solutions
- ☐ Ecosystem services
- ☐ Degrowth (reduction of global production and consumption)
- ☐ Circular use of resources
- ☐ Sustainable land management
- ☐ Technological advancement and digitalisation
- ☐ Economic prosperity
- ☐ Local and traditional food movements (e.g. slow food)
- ☐ Community resilience
- ☐ Carbon neutrality
- ☐ Sustainable consumption
- ☐ Use of biomass for multiple purposes
- ☐ Other (please specify)

[ ]

10. Which of the following sectors do you understand to be part of the bioeconomy?

- [ ] Agriculture
- [ ] Forestry
- [ ] Textiles (fashion)
- [ ] Biotechnology and pharma
- [ ] Fisheries and aquaculture
- [ ] Waste management
- [ ] Chemistry
- [ ] Construction
- [ ] Energy
- [ ] Tourism and recreation
- [ ] Machine industry
- [ ] Food and gastronomy
- [ ] Health and wellbeing
- [ ] Other (please specify)

11. To the best of your knowledge, which of the following statements about the bioeconomy are TRUE or FALSE?

	True or false?
The bioeconomy contributes to sustainable economic growth	
The bioeconomy helps mitigate the effects of climate change	
The bioeconomy contributes to the creation of new jobs	
The bioeconomy helps reduce our dependency on fossil fuels	
The bioeconomy will significantly stress natural systems	
Bio-based products are easily recycled into new products and materials at their end-of-life	
The bioeconomy contributes to deforestation	
The bioeconomy provides benefits to rural areas	
The bioeconomy provides benefits to urban areas	
The bioeconomy is a good idea, but there is not enough biomass to implement it	
The bioeconomy provides business and innovation opportunities	

Bioeconomy Perceptions Survey
Part I: Bioeconomy as a concept, benefits and risks

12. To your best knowledge, which of the following benefits of the bioeconomy is most important?

- ◯ Helping conserve biodiversity and ecosystem services
- ◯ Providing renewable alternatives to non-renewable materials
- ◯ Renewable energy to replace fossil fuels
- ◯ Job creation and economic growth
- ◯ Transition to a low-carbon economy
- ◯ Reduced material consumption and waste, increased reuse and recycling
- ◯ Fostering technological innovation
- ◯ Providing wellbeing for people
- ◯ None of the above

13. To your best knowledge, which of the following risks of the bioeconomy is most important?

- ◯ Impacts on ecosystem services
- ◯ Higher cost of essential goods (e.g. food, water, shelter, etc.)
- ◯ Increasing biomass/energy costs
- ◯ Not enough biomass to supply the bioeconomy
- ◯ Increased imports of biomass to Europe may have dangerous impacts on developing countries
- ◯ Impacts on poverty or food sovereignty
- ◯ None of the above

BIOREGIONS
Forest bioeconomy in action

Bioeconomy Perceptions Survey

Part II: Bioeconomy in (REGION)

Promising sectors

* 14. The bioeconomy is related to many different new and existing sectors.

Of the following sectors, please choose up to three which you think have the highest potential for growth in (REGION).

- ☐ Wood construction
- ☐ Green chemistry
- ☐ Bioenergy
- ☐ Nature-based tourism
- ☐ Pulp and paper
- ☐ Bioplastics
- ☐ Textiles and fashion
- ☐ Advanced new materials (for manufacturing)
- ☐ Food and gastronomy
- ☐ Non-wood forest products, (e.g. mushrooms, resins)
- ☐ Wood products, wood-based materials, and furniture
- ☐ Other (please specify)

____

Bioeconomy Perceptions Survey
Part II: Bioeconomy in (REGION)

Supporting conditions and barriers

Below we have listed potential supporting conditions and barriers to bioeconomy development in (REGION). Please evaluate the importance of each based on your opinion.

15. How important are the following factors as supporting conditions for bioeconomy development in (REGION)?

	Not at all important	Low importance	Neutral	Important	Extremely important
Public procurement programmes, to stimulate demand	○	○	○	○	○
Public/private investment in innovation	○	○	○	○	○
Availability of scientific information for better informed public and policymakers	○	○	○	○	○
Adequate regulation to overcome possible negative impacts on ecosystems and local communities	○	○	○	○	○
Performance-based payments for carbon sequestration	○	○	○	○	○

16. How important are the following factors as barriers to bioeconomy development in (REGION)?

	Not at all important	Low importance	Neutral	Important	Extremely important
Lack of profitability and market demand for bioeconomy businesses and products	○	○	○	○	○
Lack of general social acceptance	○	○	○	○	○
Lack of technical feasibility and/or barriers to innovation	○	○	○	○	○
Lack of co-operation among different stakeholders (e.g. policymakers, business, research)	○	○	○	○	○
Lack of balance between different uses of forest (e.g. economic, conservation, carbon sequestration, etc)	○	○	○	○	○
Lack of supportive policy and legislative environment well tailored to regional needs	○	○	○	○	○

BIOREGIONS
Forest bioeconomy in action

Bioeconomy Perceptions Survey
Part II: Bioeconomy in (REGION)

Roles and responsibilities

Using the sliders below, indicate whether the government or the private sector is more responsible for each condition for the development of (REGION's) bioeconomy:

17. Who is responsible for ensuring the positive environmental and social impacts of the bioeconomy?

Public sector | Both | Private sector

18. Who should invest in research, development, and innovation?

Public sector | Both | Private sector

19. Who is responsible for communicating and promoting the bioeconomy among the general public?

Public sector | Both | Private sector

BIOREGIONS
Forest bioeconomy in action

Bioeconomy Perceptions Survey
Part II: Bioeconomy in (REGION)

Common goals

20. We would like to identify overlaps between the bioeconomy and other policy areas that are important to business and government.

Please check all of the policy areas that you think are related to the aims of the bioeconomy:

- ☐ Rural development
- ☐ Clean energy
- ☐ Job creation
- ☐ Circular economy
- ☐ Technological innovation
- ☐ Biodiversity conservation
- ☐ Other (please specify)
- ☐ Parks and recreation
- ☐ Public health and wellbeing
- ☐ Urban planning
- ☐ Social inclusion
- ☐ Climate change mitigation and adaptation

[ ]

BIOREGIONS
Forest bioeconomy in action

Bioeconomy Perceptions Survey
Part II: Bioeconomy in (REGION)

Willingness to engage

21. Using the slider below, indicate how willing your industry sector would be to developing the bioeconomy:

Not willing — Neutral — Very willing

22. Has the firm where you are employed undertaken any investment projects in the bioeconomy?

- ◯ Yes
- ◯ No

23. If yes, please choose one or more reason(s):

- ☐ To gain a competitive advantage in future markets
- ☐ To take advantage of the existing market opportunities
- ☐ To take advantage of government incentives
- ☐ Other (please specify)

24. If no, please choose one or more reason(s):

- ☐ Unprepared market: too small and growing too slow
- ☐ High uncertainty in bioeconomy projects
- ☐ Low expected profitability
- ☐ Lack of technical capacity
- ☐ Other (please specify)

BIOREGIONS
Forest bioeconomy in action

Bioeconomy Perceptions Survey
Part II: Bioeconomy in (REGION)

Willingness to engage

25. Using the slider below, indicate how willing your government department would be to developing the bioeconomy:

Not willing — Neutral — Very willing

26. Has your team undertaken any regulation or initiative related to the bioeconomy?

- ◯ Yes
- ◯ No

27. If yes, please choose one or more reason(s):

- ☐ To improve availability and access to biological resources
- ☐ To ensure sustainability or social equity
- ☐ To generate markets and social acceptance of bioeconomy products
- ☐ Other (please specify)

28. If no, please choose one or more reason(s):

- ☐ Regional development plans do not promote the bioeconomy
- ☐ High uncertainty of bioeconomy outcomes
- ☐ It is not in the best interest of local strategies and policies
- ☐ Public is not asking for this
- ☐ Lack of technical capacity
- ☐ Other (please specify)

BIOREGIONS
Forest bioeconomy in action

Bioeconomy Perceptions Survey

Part II: Bioeconomy in (REGION)

Public awareness

29. In your opinion, is the general public sufficiently informed on the bioeconomy in (REGION)?

- ◯ The public is sufficiently informed
- ◯ The public is underinformed
- ◯ I don't know

Bioeconomy Perceptions Survey
Closing questions

30. Please share any further reflections you have on the bioeconomy in (REGION).
